# A systematic review of prediction models for risk of breast cancer

**DOI:** 10.1186/s12885-025-14990-4

**Published:** 2025-10-27

**Authors:** Federica Re, Natnicha Manaboriboon, Iwan G. A. Raza, Alexandra Shipley, Lucy E. Thompson, Catherine Tiplady, Marina Blum, Gina Blum, Rumbidzai Mucheke, Roxanna E. Abhari, Christiana Kartsonaki

**Affiliations:** 1 Clinical Trial Service Unit & Epidemiological Studies Unit (CTSU), Nuffield Department of Population Health, University of Oxford, Old Road Campus, Oxford, OX3 7LF UK; 2 Medical Sciences Division, University of Oxford, Oxford, UK; 3 Faculty of Medicine, Siriraj Hospital, Mahidol University, Bangkok, Thailand; 4 University of Colorado, Boulder, USA; 5 Oxford University Hospitals, NHS Foundation Trust, Oxford, UK

**Keywords:** Breast cancer, Risk prediction models, Risk assessment tools, Breast cancer screening

## Abstract

**Background:**

Predicting the risk of breast cancer (BC) can help with early diagnosis, treatment, and prevention strategies. Existing BC risk prediction models typically utilize demographic, genetic, and/or imaging-derived variables. This systematic review summarizes all developed models for BC risk in general and high-risk populations, and their discriminatory ability and calibration.

**Methods:**

MEDLINE and Embase were searched for studies developing and/or validating models estimating risk of developing BC in women and/or men. After removal of duplicates, 9,511 titles and abstracts were screened, yielding 360 full-text reviews. The current review focuses on all studies which developed a new model to predict BC risk in general and high-risk populations.

**Results:**

A total of 107 studies developing new models for BC risk prediction were included in this review. Sample sizes ranged from 535 to 2,392,998 for cohort studies, and from 133 cases with 113 controls to 95,075 cases with 75,017 controls for case–control studies. Areas under the receiver-operating characteristic curves (AUCs) ranged from 0.51 to 0.96. For studies reporting calibration using observed/expected events (O/E) ratio (*n* = 8), the range was 0.84 to 1.10. External validation was reported in 18 studies.

**Conclusions:**

Most BC risk models were developed in Caucasian populations, and their predictive ability and quality varied. Models including both demographic and genetic or imaging/biopsy data performed better than models based on demographic variables alone. However, their performance was not further improved by the addition of multiple types of such data.

**Supplementary Information:**

The online version contains supplementary material available at 10.1186/s12885-025-14990-4.

## Introduction

Globally, breast cancer is the most common malignancy, with survival strongly related to stage at diagnosis [1]. Breast screening is a widely implemented public health intervention aimed at reducing mortality through earlier diagnosis and treatment of smaller and asymptomatic cancers. In the United Kingdom, screening women in the general population between ages 50 and 70 every three years using mammography reduces breast cancer mortality [2]. However, the likelihood that a woman will benefit from screening mammography depends on her lifetime risk of developing breast cancer. Taking individual risk factors beyond age into account enables stratification of women into groups at varying risk of breast cancer. Population stratification based on predicted risk can help in early detection and prevention strategies by identifying high-risk groups that may need to be screened earlier or more frequently or referred to genetic counselling. It may also encourage participation in screening, as well as motivate lifestyle changes aimed at reducing risk of the disease.

Many risk prediction models that include characteristics such as demographic variables, menopausal status, and family history of breast cancer, have been developed and validated [3, 4]. While certain risk factors included in the models, such as the number of relatives with certain types of cancers are used clinically, in particular for individuals at a high risk, the use of risk prediction models at a population level may have limitations related to generalizability and utility [5–7]. The nature of breast cancer, with a long latency period and potential for recurrence decades after an early-stage diagnosis, complicates reliable assessment of the direct benefits of screening or preventative interventions. Nevertheless, the increased availability of testing for high-penetrance pathogenic mutations in BRCA1 and BRCA2 and other genes, and the discovery of single-nucleotide polymorphisms (SNPs) which collectively explain a substantial proportion of risk, have revived hopes of using more personalized breast cancer risk prediction models within routine screening programmes and in primary care [8, 9]. Risk prediction models may also help individuals understand their risk and make decisions on preventative interventions.

Despite these recent advances, the predictive value of breast cancer risk prediction models has not been comprehensively summarized in the literature [3, 4, 10, 11]. This systematic review summarizes the performance of risk prediction models which estimate risk of primary breast cancer in general and high-risk populations. Compared to previous literature reviews on this topic, the present study is more comprehensive, and includes more than double the number of models developed for general and high-risk populations. In addition, the present study categorizes the predictive ability of models based on the type and complexity of information used, such as genetic, clinical and lifestyle factors, and the complexity of these models, ranging from simple models using a few variables to complex models incorporating multiple data types. The inclusion of a broader range of models, coupled with a quality assessment, provides a more extensive overview of the existing methodologies and highlights the diversity and evolution of breast cancer risk prediction models. This comprehensive approach allows for a better understanding of the current landscape, identifying both commonly used and novel predictive factors, which can inform future research and model development.

## Methods

### Search strategy

We performed a systematic literature review following the Cochrane Collaboration methods and adhering to the PRISMA reporting recommendations [12, 13]. A predetermined review protocol was registered in the PROSPERO database (CRD42022300001). Ethical approval was not required for the present systematic review as it is assumed that all published research within the review will have fulfilled respective ethics standards at time of publication.

MEDLINE and Embase were searched combining terms relating to diagnosed breast cancer, including ductal carcinoma in situ and lobular carcinoma in situ, and risk prediction models. Further terms were included to select for language, study design and cohort. The literature was retrieved by using a combination of controlled vocabulary and keyword search terms, which were adapted to the requirements of each database. Details of the search strategy for MEDLINE and Embase are shown in the *Data Supplement (Supplemental Methods)*. The search was limited to studies conducted on adult participants (≥ 18 years) and published in English between database inception and 1 st February 2022.

### Eligibility criteria

Eligible studies either developed or validated a model to estimate the risk of breast cancer in women and/or men of general or high-risk populations, including newly occurring primary ipsilateral or bilateral cancer. Included models comprised of more than one risk factor for breast cancer and reported details on the development of the risk prediction model. Case–control and cohort studies were included. A minimum sample size of 500 participants for cohort studies and a minimum of 100 incident events for case–control studies also formed part of the inclusion criteria, as lower numbers are likely not appropriate for the development of a reliable risk prediction model [14]. Studies not originally published in English, as well as screening or detection (cross-sectional) studies, systematic reviews, and conference proceedings were excluded. Finally, studies that reported risk of local recurrence were excluded. Given the large volume of studies and to aid appraisal of results, the present review focuses on studies that developed a new model. Modified models and validation studies will be reviewed separately.

### Study selection and data extraction

**Fig. 1:**
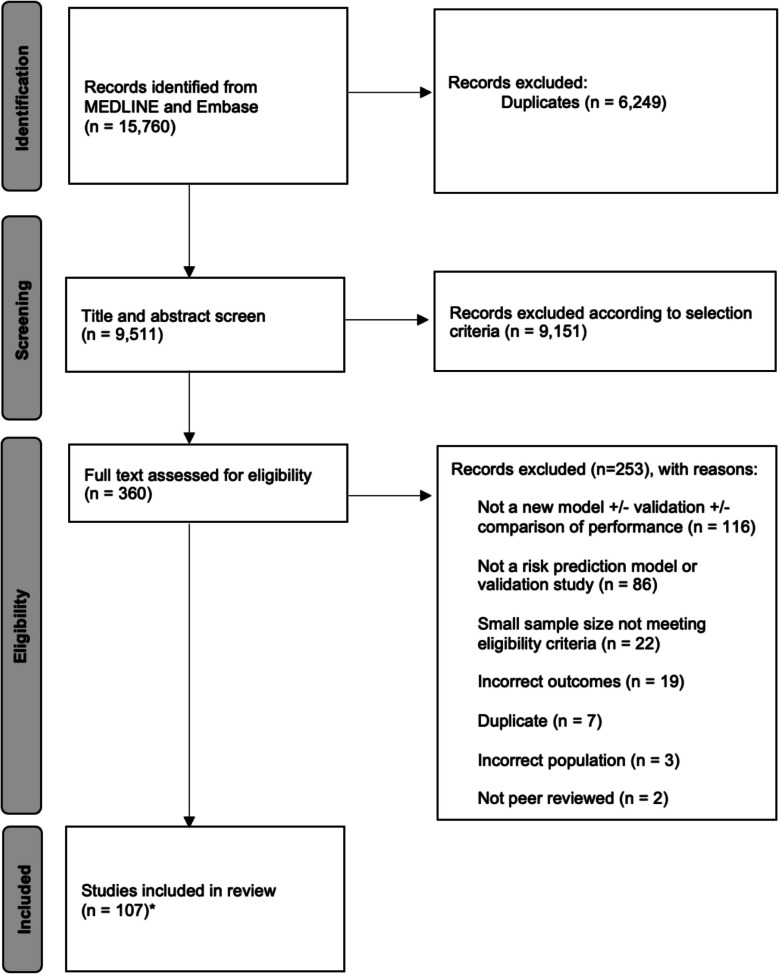
PRISMA flowchart describing the systematic literature search and study selection. *Included studies developed a novel breast cancer risk prediction model.

Studies identified from the initial search were imported into Covidence and duplicates were removed. Titles and abstracts were reviewed independently by two reviewers (FR and CK). Studies that did not meet the inclusion criteria were excluded. The full texts of the remaining papers were reviewed independently by FR and REA. Irrelevant studies were removed, and in case of disagreement between reviewers, the inclusion of studies was determined by consensus. The results of this process are detailed in a PRISMA flowchart (Fig. 1). A full list of included studies, detailing the variables included in each prediction model, has been reported in the *Data Supplement (Table ST1)*. Data extraction was conducted twice by two separate, blinded reviewers (NM, IGAR, AS, LET, CT, RM). The two extractions were subsequently compared and merged by FR, and discrepancies between extractions were checked against the full-texts and resolved accordingly. A standardized form was used to extract relevant study data, including author, publication date, name of the model if applicable, outcome type, sample characteristics, sample size, variables included in the risk score, and details of discrimination and calibration of the models. A quality assessment of all included studies was performed by two separate reviewers (MB, GB).

### Quality assessment

The Prediction Model Risk Of Bias Assessment Tool (PROBAST) was used to assess the risk of bias and applicability of models [15]. This tool assesses four domains for potential risk of bias: participants, predictors, outcome, and analysis. The tool assigns a low risk of bias rating to studies which use a robust study design, clearly define outcomes, predictors, and time frame and validate their models, and provide acceptable measures of discrimination and calibration. We aimed to provide a comprehensive summary of all breast cancer risk prediction models that have been reported in the literature, and collate as much information as possible on models and their characteristics, as well as assess the quality of studies in which models were developed.

### Data synthesis and analysis

The discriminative ability of models and calibration estimated for the specified population were extracted. Where applicable, the area under the receiver operating characteristic curve (AUROC or AUC) or C-statistic and the observed to expected (O/E) or expected to observed ratio (E/O) of events were included. Findings are reported as a narrative synthesis and results are presented according to the original model reported. The forest plots summarizing discriminatory accuracy (AUC) and their 95% confidence intervals (95% CIs) were divided into three categories (demographic, genetics, and imaging/biopsy) based on the variables included in the risk prediction models. They were further subdivided by population type (general or high-risk). The population was classed as high-risk in instances of previous breast cancer, previous cancer, known pathogenic variants (such as BRCA1 or BRCA2 mutations), or of a first degree relative with breast or ovarian cancer. For studies reporting more than one model, only the highest-performing model was displayed, with additional models reported in the *Data Supplement (Figures SF1, SF2 and SF3)*.

## Results

### Study inclusion

The initial search yielded 9,511 studies after duplicate removal. Of these, 107 developed a new model and are therefore included in this review. Figure 1 shows the number of papers excluded at each stage of the review process. At the screening stage, studies were excluded with a sample size below 500 participants for cohort studies and less than 100 incident events for case–control studies, as well as studies assessing risk of breast cancer recurrence. On full-text assessment, studies (*n* = 86) were excluded that did not develop a risk model and instead only looked at the association of a single variable with breast cancer risk, followed by studies (*n* = 22) in which the sample size or number of events did not meet the inclusion criteria. From the included studies, cohort study design was the most common, followed by case–control and nested case–control designs. The number of participants varied between 535 and 2,392,998 for cohort studies, and 133 cases with 113 controls and 95,075 cases with 75,017 controls for case–control studies. There were 41 (38.3%) studies conducted in the USA [16–57], 13 (12%) in the United Kingdom, 11 (10.2%) in China [58–68], 3 (2.7%) in Japan [69–71], and 3 (2.7%) in Canada [72–74]. Of the remaining 36 studies, 18 were conducted across Europe (Cyprus [1] [75], Finland [1] [76], Germany [3] [77–79], Greece [1] [80], Italy [1] [81], The Netherlands [3] [82, 83], Slovakia [1] [84], Spain [1] [85], Sweden [4] [86–89], Switzerland [2] [90]), 9 in Asia (Hong Kong [1] [91], Iran [1] [92], Saudi Arabia [2] [93, 94], Singapore [2] [95, 96], South Korea [1] [97], Taiwan [1] [98], Thailand [1] [99]), two in Australia [100, 101], and one in Africa (Nigeria) [102]. There were eight studies developed using data from multiple countries, with five across multiple European countries [62, 103–106], one across multiple Asian countries [107], and two across continents [108, 109]. A total of 68 studies focused on predicting Lifetime breast cancer risk, while other outcomes included: 5-year breast cancer risk (*n* = 19), 10-year breast cancer risk (*n* = 17), short-term risk, meaning risk of diagnosis within the following three years or less (*n* = 6), overall risk by a certain age cut-off, for example risk of diagnosis by e.g. age 50 (*n* = 3), oestrogen- and progesterone-receptor (ER and PR) positive breast cancer risk (*n* = 2) and contralateral cancer risk (*n* = 1), with several studies assessing more than one outcome.

### Study characteristics

*Table ST1 (Data Supplement)* illustrates the characteristics of the 106 included studies. A total of 89 studies constructed models in the general population, while 18 studies focused on high-risk populations. Risk prediction models included up to 22 variables. For studies including SNPs, these were mainly reported as polygenic risk scores (PRS) calculated using up to 3,820 SNPs [106]. Most models included age, body mass index (BMI, kg/m^2^), and family history of breast cancer. A categorized list of variables included across all risk models has been reported in the *Data Supplement (Table ST2).* A total of 112 variables were included across all models. These can be sub-divided into the following categories: reproductive history and characteristics (*n* = 25), imaging and procedural investigations (*n* = 24), demographics (*n* = 19), breast cancer history (*n* = 16), genetic information (*n* = 12), social history (*n* = 11), and family history (*n* = 6). For studies that developed demographic models, the main variables included were age, BMI, family history or breast cancer, and reproductive history characteristics. For studies that developed models using genetic data, some included PRS combining up to 3,820 SNPs [110], while others included high-penetrance pathogenic variants in genes such as BRCA1 and BRCA2. Some studies developed imaging/biopsy models, which mainly included mammographic or ultrasound scan density measures, as well as biopsy characteristics such as tumour size, histology, and nodal status. Studies were divided into three categories (demographic only, genetics, and imaging/biopsy) based on the variables included in the models. Models were grouped based on the complexity of the variables included: imaging/biopsy potentially also contained demographic variables and genetic models potentially also contained imaging/biopsy with or without demographic variables.

A total of 83 studies reported a measure of discrimination, which ranged from 0.51 to 0.96 (no 95% CIs reported for these two values) [92, 110]. A total of 20 studies reported other measures of effect, including relative risks, hazard ratios, and net reclassification index (NRI) *(Data Supplement, Table ST3)*. Only 18 studies performed external validation *(Data Supplement, Table ST4)*, though it is possible that certain studies may have published validation results separately. Calibration was only reported in a small subset of studies, using various methods. For studies reporting calibration using O/E ratio, the range was 0.84 (no 95% CI reported) to 1.10 (95% CI: 1.05–1.14) [49, 89].

### Discriminatory performance and calibration

**Fig. 2:**
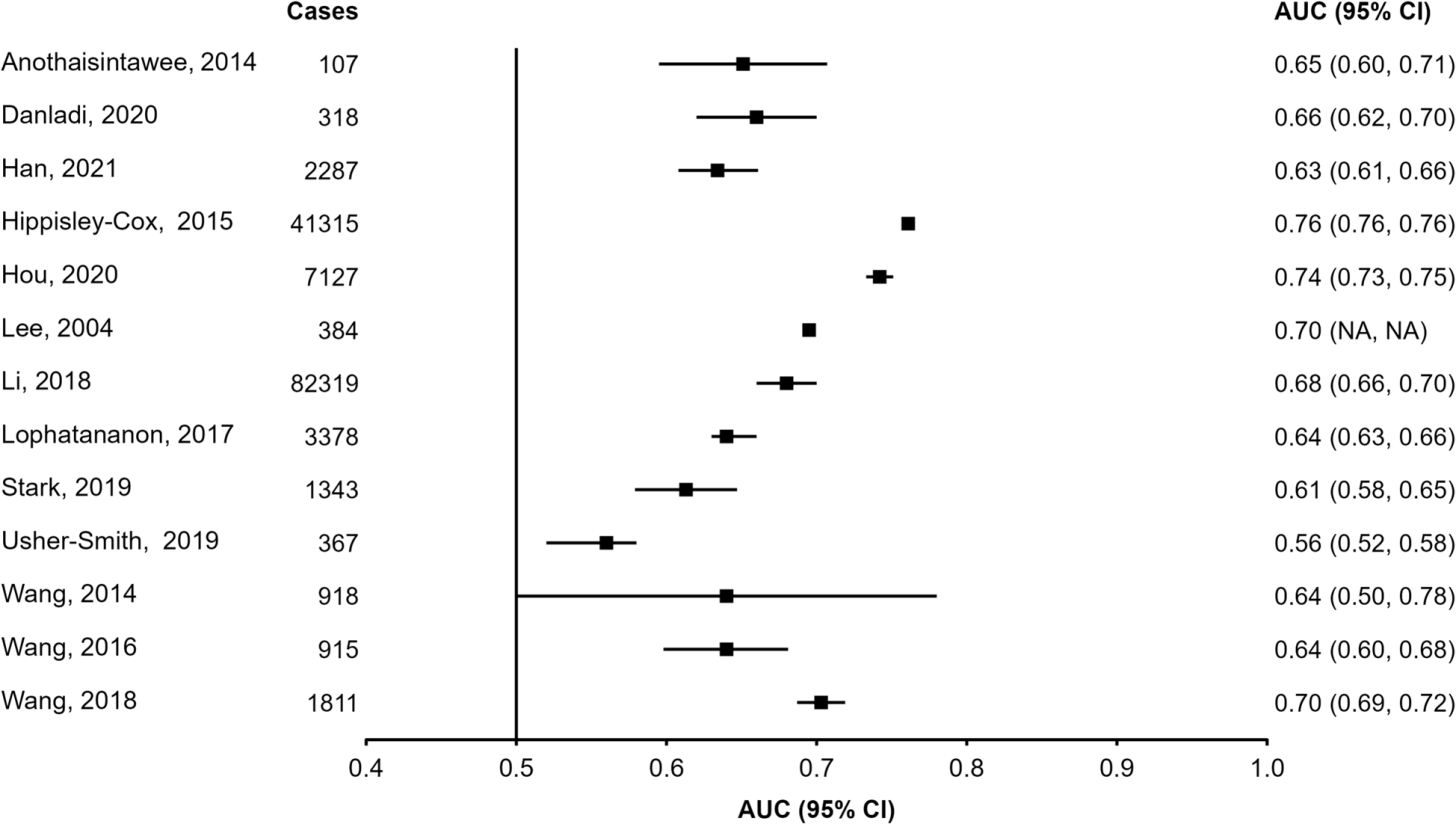
Area under the receiver operating curve (AUC) and associated 95% confidence intervals (95% CIs) for models containing demographic variables and developed in general populations. For each study, only the highest-performing model was included in the plot, with additional models reported in the Data Supplement (Figure S6). Squares represent the AUC and error bars represent the 95% CIs. NA indicates that the data were not reported.

**Fig. 3:**
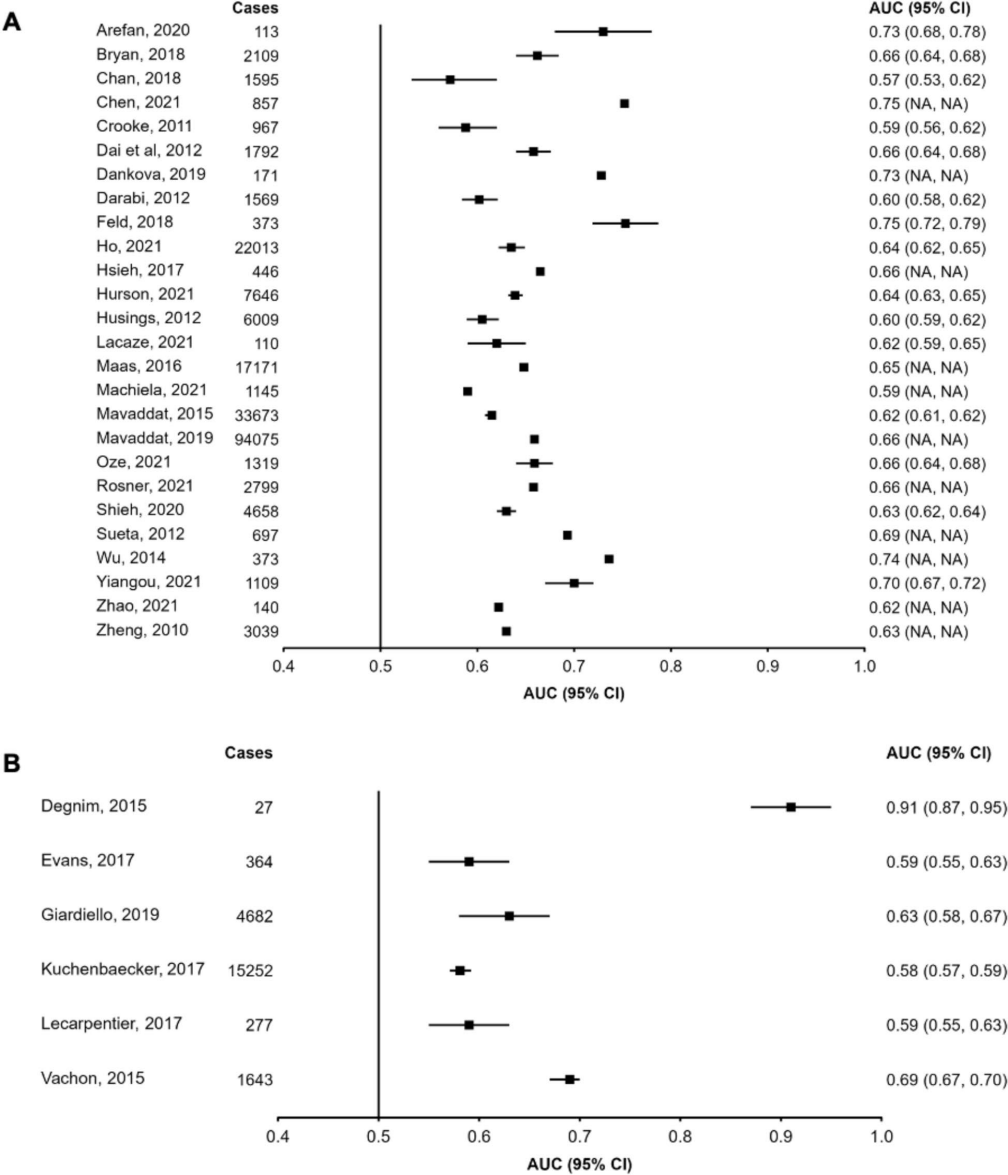
Area under the receiver operating curve (AUC) and associated 95% confidence intervals (95% CIs) for models containing genetic variables and developed in general (Panel **A**) and high-risk (Panel **B**) populations. For each study, only the highest-performing model was included in the plot, with additional models reported in the Data Supplement (Figure S6). Squares represent the AUC and error bars represent the 95% CIs. NA indicates that the data were not reported.

**Fig. 4:**
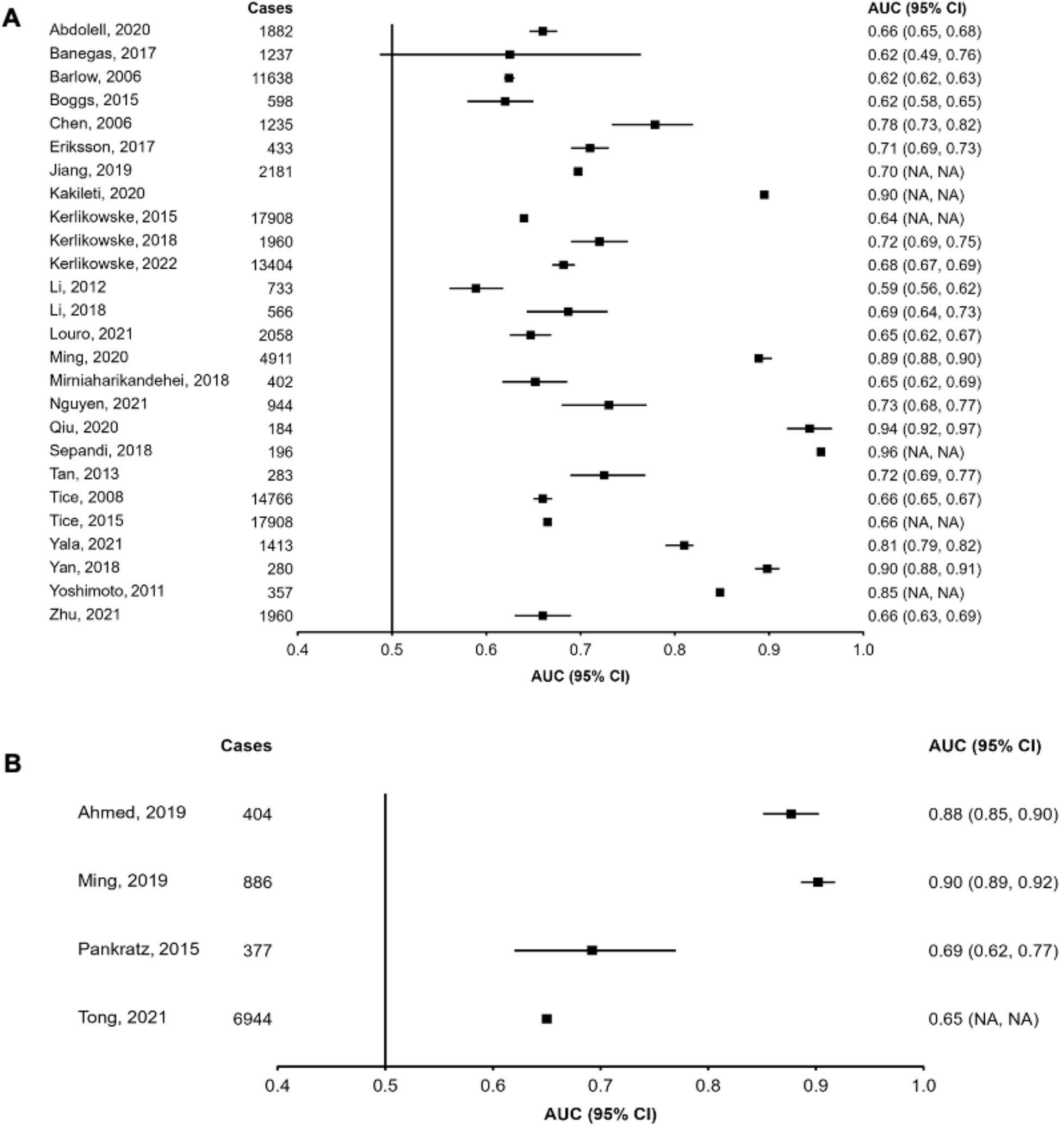
Area under the receiver operating curve (AUC) and associated 95% confidence intervals (95% CIs) for models containing imaging and biopsy variables and developed in general (Panel **A**) and high-risk (Panel **B**) populations. For each study, only the highest-performing model was included in the plot, with additional models reported in the Data Supplement (Figure S7). Squares represent the AUC and error bars represent the 95% CIs. NA indicates that the data were not reported.

Figures 2, 3 and 4 display the discriminatory accuracy and associated 95% CIs of included models reporting an AUC for general and high-risk populations. Given that the study herein does not attempt to combine estimates into a pooled effect size it was deemed reasonable to combine high-risk populations together despite differences in their definition. Only the highest performing model for each study was included in the figures, with additional model variants reported in the *Data Supplement (Figures SF1, SF2 and SF3)*. Overall, out of the studies reporting AUCs, a greater number developed models in general (*n* = 65) rather than high-risk (*n* = 10) populations. Studies based on demographic variables alone (*n*= 13) were only developed within general populations. Among general population samples, 26 studies included genetic data and 28 included imaging and biopsy information. Among high-risk populations, six studies included genetic data and four included imaging or biopsy information. Studies reporting ≥ 20,000 cases tended to favour genetics-based models, with most studies including PRS’ composed of a combination of SNPs [20, 106, 107, 110]. One study including ≥ 20,000 cases developed a model using demographic variables only, which included personal demographic characteristics, reproductive characteristics, and a variable of interaction between BMI and menopause status [62]. No studies containing ≥ 20,000 cases developed imaging and/or biopsy-based models.

AUCs were higher in models containing imaging and biopsy information, followed by models containing genetic variables, and those composed solely of demographic characteristics. Aside from the distinction between models containing solely demographic variables and those with additional data, additional model complexity did not influence results, with models containing both genetic and imaging/biopsy variables not performing substantially better than models containing either genetic or imaging/biopsy data alone. Notably, genetics and imaging/biopsy models that also contained demographic data performed better than those that did not. Larger studies reported lower AUCs and narrower 95% CIs than studies with fewer cases, which tended towards higher AUCs and a greater spread of results. The AUC range for studies with < 10,000 cases was 0.51 (no 95% CIs reported) to 0.96 (no 95% CIs reported), whereas that for studies with > 10,000 cases was 0.62 (95%CI: 0.62–0.63) to 0.68 (95%CI: 0.67–0.69) [18, 35, 92, 110]. No major difference was observed between AUCs of studies developed in general versus high-risk populations. Of the studies reporting an AUC, only 26 studies reported model calibration results, which displayed significant heterogeneity (O/E ratio range = 0.84 to 1.10; *Tables ST3 and ST4)*.

### Quality assessment

The quality of included studies was assessed using the PROBAST tool *(Data Supplement Table ST5)*. Based on the PROBAST assessment, most studies demonstrated a low risk of bias across most domains. However, concerns were more apparent in the participants and analysis domains, with several studies rated as high or unclear risk of bias. High-risk ratings in participants were often due to non-representative populations or limited inclusion criteria, while deficiencies in analysis typically related to inadequate handling of missing data, inappropriate statistical methods, or lack of model validation. Applicability concerns were generally minimal, with most studies showing low concern across participants, predictors, and outcomes.

## Discussion

This systematic review included 107 studies that developed new models aimed at estimating the risk of breast cancer in general and high-risk populations. The most widely included risk factor was age, followed by reproductive characteristics, though most models included genetic and/or imaging and biopsy variables. Discriminatory measures ranged from 0.51 to 0.96 [92, 110]. Calibration was only available for a small number of models.

There have been an increasing number of risk prediction models for breast cancer developed over the past 30 years, with current models displaying overall good predictive power. New variables, including a wider range of common genetic variants such as SNPs and BRCA1/2 pathogenic variant status, have been included in models of risk prediction, as described in other reviews [3, 4]. Compared to previous systematic reviews on this topic, the present review has wider inclusion criteria and identified more than double the number of risk prediction models among both general and high-risk populations, as well models developed in non-Caucasian ethnic groups [4, 11]. In addition, we also included models containing tumour-associated antigens and biomarkers and models developed in high-risk populations [38, 55, 64, 87].

Several models demonstrated good discriminatory capability. Adding variables such as SNPs, mammographic density, and tumour-associated antigens to risk prediction models generally improved performance, though this remained highly variable across models. However, performance is likely to depend largely on the population in which the risk model was developed and tested in. Most of the models were developed and validated in general Caucasian populations, so their applicability and performance in other ethnic groups, or indeed in high-risk populations, remains under-explored [30, 110, 111]. Given the variation in AUCs reported across all three main types of models and the heterogeneity of populations these were developed in, it remains unclear whether specific model types differ significantly in performance. Recent breast cancer risk prediction models often utilise PRS and mammographic density, either alone or in combination. Increasingly, models which included mammographic density or PRS alone appeared to perform similarly, with models combining both variable types not performing notably better [112]. Studies with a larger patient cohort appeared to report smaller AUCs compared to studies with fewer cases. This phenomenon can occur due to overfitting of the predictive model, which occurs when complex models are fit too closely to a training dataset, limiting generalizability [113]. Equally, however, larger cohorts are more likely to be representative of the general population, thus smaller AUCs may be both expected and more representative of the true population risk. Overall, while a large proportion of studies achieved low risk of bias and high applicability in quality assessment, methodological weaknesses in analytical rigor were the most frequent limitations, suggesting these areas should be prioritized for improvement in future model development and validation studies.

The format of risk prediction models is also crucial for their application in clinical settings. Relative risk and absolute risk provide different but complementary insights. Relative risk indicates how much more likely an individual with certain risk factors is to develop breast cancer compared to someone without those risk factors. This can be useful for identifying high-risk individuals but may be less intuitive for patients and clinicians when making specific healthcare decisions. Absolute risk, on the other hand, provides the probability that an individual will develop breast cancer over a specific time period. This format is often preferred in clinical settings as it offers a clearer understanding of an individual's risk, facilitating informed decision-making and personalized risk management strategies. Future studies should aim to present both relative and absolute risks to enhance the utility of risk prediction models in clinical practice.

Other factors that may have affected the predictive ability of models include data availability, sample Heterogeneity, choice of variables, and choice of analysis method. Finally, only 18 studies in this review presented results of external validation, although this data may have been published separately. The true value of risk prediction models is best assessed through external validation using independent datasets, which typically includes both discrimination and calibration assessments. The lack of external validation or calibration in the majority of included studies therefore underscores a significant gap in the current literature, though some studies assessed validation separately. Mis-calibrated models can lead to inaccurate risk predictions, where the predicted risks do not match the observed outcomes, potentially leading to inappropriate clinical decisions. AUC values derived from the same data used to develop the models can be overly optimistic and may not reflect the models' performance in real-world settings. Consequently, while studies without external validation or calibration contribute to the development of predictive models, their ability to determine the true value of risk predictions is limited. Future research should prioritize external validation and calibration assessment to ensure that models are both accurate and reliable in diverse populations.

It is important to consider the practicality of using risk prediction in clinical settings. For example, the inclusion of genetic markers, such as BRCA1 and BRCA2, may improve breast cancer risk prediction but may be unfeasible to deploy in population-wide screening programs due to costs, lab-related analysis burden, ethical concerns, and time delays. How these models fit within clinical practice also requires consideration given the potential requirements for patient counselling. Models aimed at high-risk individuals are more likely to be useful in practice given this is an area not covered by population-level screening strategies, but equally this poses questions around the implications on decision about possible preventative treatment, including prophylactic mastectomies and oophorectomies or oestrogen modulating therapies, for high-risk groups.

Another factor to consider is the feasibility of identifying risk factors necessary for the models. Risk factors become apparent at different stages in life. For example, risk assessment based on lifestyle factors and/or mammographic density can only be performed when an individual has reached the fourth or fifth decade of life, which is often too late to predict premenopausal breast cancer. Contrastingly, germline genetic variants can be measured from birth. While an ethical discussion of these strategies is beyond the scope of this review, the value of estimating a person’s risk in different circumstances requires consideration. Equally, the varying complexity of the risk prediction models versus the complexity of an individual’s risk merits consideration. Genetics-based models often include the highest complexity of information, but obtaining, storing, and computing this information may not be straightforward, nor applicable to all patient cohorts. For example, there might be limited value in utilizing a more complex model versus a simpler one (e.g., genetic versus demographic models) in patients who are not at high risk of breast cancer based on known baseline characteristics, such as family history. Whether more complex risk prediction models may be applied among individuals meeting some initial screening criteria, or whether the models could replace these criteria in the first place, is being explored, with several models such as Gail and BOADICEA/CanRisk already being used in clinical practice [8, 114].

The current review has several strengths. Firstly, the broad inclusion criteria and large number of models included make this review the most comprehensive and largest study summarizing breast cancer risk prediction models to date, and may serve as a reference for future studies aiming to evaluate the performance of models. Secondly, full-text screening and data abstraction was performed by multiple, blinded, reviewers which increases the quality of the review process. With regards to limitations, validation was only included if reported within the same paper, but some studies may have published this separately. However, given the comprehensive search in Medline and EMBASE, it is estimated that the loss of information due to these factors is low.

We exclusively included case–control and cohort studies due to their suitability for evaluating breast cancer risk prediction models. These study designs provide longitudinal data and allow for systematic comparisons, which is essential for developing and validating robust predictive models. However, other aspects of the study design, such as matching in case–control studies, or age, can influence the comparability of the AUC values. Matching may lead to higher AUCs by reducing the variability of matched factors, thus enhancing the apparent predictive performance of models. Similarly, although our review sought to provide a comprehensive evaluation of breast cancer risk prediction models, it includes only one study examining transcriptional profiles and excludes studies that developed models purely theoretically without population-based validation, such as Tyrer et al*.,*2004 [115]. This selection was based on our inclusion criteria, but future research could include OMIC-based models as this is a rapidly evolving field with the potential to significantly enhance predictive accuracy and personalized care. Equally, future research extending the present review will also consider studies that, while not developing novel models, have evaluated existing models across different populations and settings.

## Conclusion

This review presents the largest and most comprehensive review of breast cancer risk models to date. The 107 breast cancer risk prediction studies included performed variably. Models that included demographic data and other type of data (imaging/biopsy or genetic data) performed better than models that included solely demographic data. However, their performance was not better when of multiple types of such data were included. The question of whether the inclusion of secondary care data in the risk prediction models would be cost-effective for the general population, or should be limited to a high-risk group, remains unexplored. Identifying high-risk individuals accurately requires balancing sensitivity, specificity, and cost-effectiveness, and it might be naïve to use the best model if it is too costly or impractical for widespread use. Although the inclusion criteria were broad, most models were developed in general Caucasian populations, so the applicability of these models to other ethnicities is an area of further research.

## Supplementary Information

Supplementary Material 1.

## Data Availability

All data generated or analysed during this study are included in this published article and its Supplementary information files.

## Abbreviations

AUC: Area under the curve
AUROC: Area under the receiver-operating characteristic curve
O/E: Observed over expected ratio
E/O: Expected over observed ratio
SNPs: Single-nucleotide polymorphisms
95%CI: 95% Confidence interval
PRS: Polygenic risk score
BMI: Body mass index, kg/m^2^
NRI: Net reclassification index
