# Supplementary material for: A systematic review of prediction models for risk of breast cancer
Source: BMC Cancer. 2025 Oct 27;25:1650. doi: 10.1186/s12885-025-14990-4 (PMC12560532; doi:10.1186/s12885-025-14990-4)
Supplement: Supplementary file 1 — Supplementary Material 1. [file 12885_2025_14990_MOESM1_ESM.pdf]

A Systematic Review of Prediction Models for Risk of Breast Cancer

DATA SUPPLEMENT

Federica Re [1,2]
Natnicha Manaboriboon [3]
Iwan G. A. Raza [2]
Alexandra Shipley [2]
Lucy E. Thompson [2]
Catherine Tiplady [2]
Marina Blum [2]
Gina Blum [4]
Rumbidzai Mucheke [5]
Roxanna E. Abhari† [1,2]
Christiana Kartsonaki† [1]

† *Joint Senior Authorship*

[1] Clinical Trial Service Unit & Epidemiological Studies Unit (CTSU), Nuffield Department of Population Health, University of Oxford, Oxford, UK.

[2] Medical Sciences Division, University of Oxford, Oxford, UK.

[3] Faculty of Medicine, Siriraj Hospital, Mahidol University, Bangkok, Thailand.

[4] University of Colorado, Boulder, United States.

[5] Oxford University Hospitals, NHS Foundation Trust, Oxford, UK.

Address for correspondence
Dr Christiana Kartsonaki
CTSU, Nuffield Department of Population Health
University of Oxford
Old Road Campus
Oxford, OX3 7LF, UK
christiana.kartsonaki@dph.ox.ac.uk

Table ST1. Summary of included breast cancer risk prediction models across 107 included studies. Abbreviations: USA=United States of America; UK=United Kingdom; BC=breast cancer; BMI=body mass index; DCIS=ductal carcinoma in situ; ER=estrogen receptor; FHx=family history; GRS=genetic risk score; GWAS=genome-wide association study; HER=human epidermal growth factor receptor2; HRT=hormone replacement therapy; IGF1=Insulin-like growth factor; OCP=oral contraceptive pill; PR=progesterone receptor; PRS=polygenic risk scores; SERMs=selective estrogen receptor modulators; SNPs=single nucleotide polymorphisms; WHR=weight to hip ratio; WHtR=waist to height ratio.

First author, year	Country	Outcome	Population type	Data source	Factors included in risk score
Abdolell, 2020 (Imaging)	Canada	BC risk	General	Medical records	Age, mammographic density, breast volume
Abdolell, 2020 (Imaging + core biopsy)					Age, mammographic density, breast volume, biopsy
Abdolell, 2020 (Imaging + core biopsy + FHx)					Age, mammographic density, breast volume, biopsy, 1st degree relative with BC
Abdolell, 2020 (All factors)					Age, mammographic density, breast volume, biopsy, 1st degree relative with BC, parity, OCP
Ahmed, 2019	Saudi Arabia	BC risk	High-risk: symptomatic women who underwent a breast mass biopsy	Medical records	Age, family history of BC, HRT, OCP, mammographic density, ultrasound density, biopsy, menopausal status
Anothaisintawee, 2014	Thailand	BC risk	General	Interviews, medical records	Age, BMI, OCP, menopausal status
Antoniou, 2002	UK	BC risk by age 70	General	Anglian BC Study	Age, family history of BC, 1st degree relative with BC, BRCA 1/2, history of ovarian cancer, family history of ovarian cancer
Arefan, 2020	USA	Short term BC risk	General	Medical records	Age, family history of BC, 1st degree relative with BC, BRCA 1/2, mammographic density
Arthur, 2020	UK	Invasive BC risk	General	UK biobank	Age, socioeconomic status, BMI, alcohol, smoking history, family history of BC, age at menarche, age of menopause, age at first live birth, parity, HRT, OCP, SNP, GRS, PRS, physical activity, diet
Babiker, 2020	Saudi Arabia	BC risk	General	Medical records, interview	Age, BMI, family history of BC, age at first live birth, parity, breastfeeding, menopausal status, marital status
Banegas, 2017	USA (Hispanic)	BC risk	General	Medical records	Age, family history of BC, age at menarche, age at first live birth, benign breast disease, biopsy
Barlow, 2006	USA	Short-term BC risk	General	Breast Cancer Surveillance Consortium (BCSC)	Age, BMI, family history of BC, 1st degree relative with BC, age at menarche, age at first

					live birth, HRT, mammographic density, prior breast procedure
Barnes, 2020	29 European countries	10-year BC risk	High risk: BRCA1/2	Consortium of Investigators of Modifiers of BRCA1/2	Family history of BC, BRCA1/2, SNPs
Behravan, 2020	Finland	BC risk	General	Kuopio Breast Cancer Project database	Age, BMI, family history of BC, 1st degree relative with BC, parity, breastfeeding, HRT, OCP, SNPs, menopausal status, pregnancy length, menstrual cycle length, laterality of cancer in family
Boggs, 2015	USA (African American)	5-year BC risk	General	Black women's health study	BMI, height, family history of BC, 1st degree relative with BC, age at menarche, age at first live birth, HRT, OCP, benign breast disease, biopsy, bilateral oophorectomy
Borde, 2021	Germany	BC risk	High-risk: CHEK2 gene mutation carriers	German Consortium for Hereditary Breast and Ovarian Cancer database	Age, SNPs, CHEK2 gene mutation
Bryan, 2018 (Whole-genome linear mixed model)	USA (European ancestry women)	BC diagnosed before 50 years old	General	Breast Cancer Family Registry (BCFR) , the German Genetic Epidemiologic Study of Breast Cancer, the Long Island Breast Cancer Study Project, the Seattle Study, and the Chicago Multi-ethnic Breast Cancer Epidemiologic Cohort	Whole genome
Bryan, 2018 (Polygenic risk scores, PRS)					PRS
Bryan, 2018 (Non-genetic epidemiologic factors)					Age, ethnicity, education, marital status, smoking history, age of menarche, parity, OCP
Bryan, 2018 (Non-genetic epidemiologic factors + whole-genome linear mixed model)					Age, ethnicity, education, marital status, smoking history, age of menarche, parity, OCP, whole genome
Chan, 2018	Singapore (Singaporean Chinese)	BC risk	General	Medical records	Age, SNPs
Chen, 2006	USA	5-year BC risk	General	Breast Cancer Detection Demonstration Project (BCDDP)	Age, weight, 1st degree relative with BC, age at first live birth, mammographic density, biopsy
Chen, 2021	China	3-year and 5-year BC risk	General	TCGA database	Immune genes
Chowdhury, 2017	USA	Contralateral BC risk	High risk: unilateral BC	Breast Cancer Surveillance Consortium database	Age, BMI, family history of BC, 1st degree relative with BC, age at first live birth, mammographic density (BI-RADS), biopsy, age

					at first BC diagnosis, ER status, high risk pre-neoplasia status, anti-oestrogen therapy, type of first BC
Colditz, 1996	USA	BC risk	General	Questionnaire	Age, age at menarche, age of menopause, age at first live birth, parity, age at second/third birth
Crooke, 2011	Germany	BC risk	General	GENICA study database	Age, BMI, family history of BC, 1st degree relative with BC, age at menarche, age of menopause, parity, HRT, OCP, CYP1A1, CYP1B1 and COMT genotype
Dai, 2012	China	BC risk	General	Medical records	Age, age at menarche, age at first live birth, SNPs
Dankova, 2019	Slovakia	BC risk	General	Medical records	Age, SNPs
Danladi, 2020	Cyprus	BC risk	General	Medical records, interviews	Age, 1st degree relative with BC
Darabi, 2012	Sweden	5-year and 10-year BC risk	General	Study<br>Medical records	Age, BMI, family history of BC, age at menarche, age at first live birth, SNP, benign breast disease
Degnim, 2015	USA	10-year BC risk	High risk: sclerosing adenosis patients	Mayo BBD cohort database	Transcriptional profiles of FFPE biopsy-derived RNA
Dembrower, 2020	Sweden	BC risk	General	Medical records	Age, mammographic density
Eriksson, 2017	Sweden	BC risk	General	Karma cohort	Age, BMI, family history of BC, 1st degree relative with BC, HRT, mammographic density, computer-aided detection of microcalcifications and masses, menopausal status
Evans, 2017	UK	10-year BC risk	High risk: women with familial history of BC	Medical records	Age, WHtR, height, family history of BC, 1st degree relative with BC, age at menarche, age of menopause, age at first live birth, BRCA1/2, SNP, benign breast disease
Feld, 2018	USA	BC risk	General	Marshfield Clinic Personalized Medicine Research Project	Age, family history of BC, 1st degree relative with BC, SNP, mammographic density (mass margins, microcalcification shape, microcalcification distribution, and architectural distortion), biopsy, number of breast biopsies
Gail, 1989	USA	BC risk	High risk: women with in-situ and invasive carcinoma	Breast Cancer Detection Demonstration project	Age at menarche, number of previous breast biopsies, age at first live birth, number of first-degree relatives with breast cancer
Gao, 2021	USA	5-year BC risk, lifetime absolute BC risk	General	Cancer Risk Estimates Related to Susceptibility consortium	Age, family history of BC, 1st degree relative with BC, BRCA1/2, GRS, ATM, CHEK2, PALB2, BARD1, BRIP1, CDH1, and NF1

Giordiello, 2019 (PredictCBC-1A at 5-years and 10-years)	The Netherlands	5-year and 10-year contralateral BC risk	High risk: women with invasive non-metastatic first primary BC	Medical records, the Breast Cancer Association Consortium (BCAC), the Netherlands Cancer Registry	Age, 1st degree relative with BC, BRCA1/2, age at first BC diagnosis, nodal status, tumor size, grade, morphology, ER status, PR status, HER2 status, chemotherapy, adjuvant endocrine therapy, adjuvant trastuzumab therapy, radiotherapy
Giordiello, 2019 (PredictCBC-1B at 5-years and 10-years)					Age, 1st degree relative with BC, age at first BC diagnosis, nodal status, tumor size, grade, morphology, ER status, PR status, HER2 status, chemotherapy, adjuvant endocrine therapy, adjuvant trastuzumab therapy, radiotherapy
Gorla, 2005	USA	ER+ and PR+ BC risk	High risk: women diagnosed with BC	Breast cancer database at the Lynn Sage Comprehensive Breast Center	Age, ethnicity, BMI, alcohol, smoking history, family history of BC, 1st degree relative with BC, age at menarche, age of menopause, age at first live birth, parity, HRT, OCP, mammographic density, biopsy, ER status, PR status
Hajiloo, 2013	Canada	BC risk	General	Medical records	SNPs
Han, 2021 (CKB)	China	BC risk	General	China Kadoorie Biobank	Age, BMI, family history of BC, age at menarche, parity, residence area, education, height
Han, 2021 (SWHS)				Shanghai Women's Health Study (SWHS)	
He, 2012	Cross-European	BC risk	General	ReproGen Consortium	Age, BMI, alcohol, family history of BC, 1st degree relative with BC, age at menarche, age of menopause, age at first live birth, parity, HRT, OCP, SNPs
Hippisley-Cox, 2015	UK	BC risk	General	QResearch database	Age, BMI, Townsend deprivation index, ethnicity, alcohol, family history of breast cancer, benign breast disease, oral contraceptive pill, oestrogen containing HRT, manic depression or schizophrenia, previous blood cancer, previous lung cancer, and previous ovarian cancer
Hou, 2020 (XGBoost)	China	BC risk	General	Breast Cancer Information Management System	Age, BMI, family history of BC, 1st degree relative with BC, age at menarche, age of menopause, age at first live birth, parity, residence, menopause status, duration of reproductive life span
Hou, 2020 (deep neural network)					
Hou, 2020 (random forest)					
Hou, 2020 (logistic regression)					

Ho, 2021	Malaysia, Singapore, China (East Asian)	10-year and lifetime BC risk	General	BCAC and Asia Breast Cancer Consortium	Ethnicity, SNPs
Hsieh, 2017 (clinical risk factors only)	Taiwan	BC risk	General	Medical records	Age, BMI, age at menarche, parity, menopausal status
Hsieh, 2017 (PRS + clinical risk factors)					Age, BMI, age at menarche, parity, SNPs, menopausal status
Hughes, 2020	USA	BC risk	General	Questionnaire, medical records	Age, ethnicity, family history of BC, 1st degree relative with BC, SNPs
Hughes, 2021	USA	5-year BC risk and lifetime BC risk	General	Medical records	BMI, height, weight, family history of BC, 1st degree relative with BC, age at menarche, age of menopause, age at first live birth, parity, HRT (length and type), SNPs, menopausal status
Hurson, 2021 (iCARE-lit model ages 18-50)	USA	5-year BC risk	General	European Prospective Investigation into Cancer and Nutrition (EPIC) Cohort	Age, BMI, height, alcohol, smoking history, family history of BC, 1st degree relative with BC, age at menarche, age at first live birth, parity, OCP, SNPs, benign breast disease
Hurson, 2021 (iCARE-lit model ages >50-70)					Age, BMI, height, alcohol, smoking history, family history of BC, 1st degree relative with BC, age at menarche, age of menopause, age at first live birth, parity, HRT, OCP, SNPs, benign breast disease
Husing, 2012 (Epidemiological factors)	Germany	Invasive BC risk	General	Breast and Prostate Cancer Cohort Consortium (BPC3)	BMI, height, alcohol, smoking history, age at menarche, age of menopause, age at first live birth, parity, HRT
Husing, 2012 (GWAS- 18 SNPs)					GWAS
Husing, 2012 (GWAS-18 SNPs + epidemiological factors)					BMI, height, alcohol, smoking history, age at menarche, age of menopause, age at first live birth, parity, HRT, GWAS
Jiang, 2019 (model 1a/1b)	USA	5-year BC risk	General	The sister study	Family history of BC
Jiang, 2019 (model 2a/2b)					Family history of BC, age at menarche, total biopsies, age at first live birth, age >50 indicator
Jiang, 2019 (model 3a/3b)					Family history of BC, age at menarche, total biopsies, age at first live birth, age >50 indicator, BMI, menopause status
Jung, 2018 (GWAS SNPs + epidemiological factors)	USA (non-Hispanic White)	BC risk	High risk: postmenopausal women	Questionnaire, Women's Health Initiative (WHI) Harmonized and	Age, socioeconomic status, family income, BMI, height, alcohol, smoking history, family history of BC, age at menarche, age of menopause, parity, HRT, OCP, depressive symptoms, WHR, %

				Imputed Genome-Wide Association Studies (GWAS) data	calories from SFAs, % calories from fat, physical activity, family income, history of hysterectomy/oophorectomy, estrogen use, estrogen + progestogen use
Jung, 2018 (refined model- 3 SNPs + alcohol intake + BMI)					BMI, alcohol. SNPs
Kakileti, 2020	The Netherlands	BC risk	General	Medical records	Thermography imaging
Kerlikowske, 2015, (One BIRAD at 5-years and 10-years)	USA	5-year and 10-year BC risk	General	Breast Cancer Surveillance Consortium	1st degree relative with BC, mammographic density, biopsy
Kerlikowske, 2015, (Two BIRADs 5-years and 10-years)					
Kerlikowske, 2018	USA	5-year BC risk	General	Medical records	Age, ethnicity, BMI, 1st degree relative with BC, mammographic density (clinical BI-RADS, automatic BI-RADS), biopsy
Kerlikowske, 2022	USA	5-year BC risk	General	Medical records, Breast Cancer Surveillance Consortium	Age, ethnicity, BMI, 1st degree relative with BC, mammographic density, biopsy
Kuchenbaecker, 2017	UK	BC risk	High risk: BRCA1/2 carriers	Consortium of Investigators of Modifiers of BRCA1/2 (CIMBA)	PRS
Lacaze, 2021	Australia	BC risk	High risk	ASPREE trial database	Age, BMI, alcohol, family history of BC, 1st degree relative with BC, parity, HRT, BRCA 1/2, GRS, PNS
Läll, 2019	Estonia	BC risk	General	Estonian Biobank	Age, age at menarche, age at first live birth, GRS
	Estonia and UK	10-year BC risk		Estonian Biobank, UK biobank	Age, BMI, height, family history of BC, 1st degree relative with BC, age at menarche, age at menopause, age at first live birth, parity, BRCA1/2
Lecarpentier, 2017 (ER+ PRS)	UK	BC risk	High risk: BRCA1/2 carriers	Consortium of Investigators of Modifiers of BRCA1/2 (CIMBA)	Age, age at first BC diagnosis, BRCA 1/2
Lecarpentier, 2017 (ER- PRS)					
Lee, 2004	South Korea	BC risk	General	Questionnaire, medical records	Age, socioeconomic status, education, BMI, 1st degree relative with BC, age at first live birth, breastfeeding, menstrual regularity, total

					menstrual duration. *Different risk factors used in predictive models between two groups.*
Lee, 2014	Singapore	5-year BC risk	General	Singapore Chinese Health Study	Socioeconomic status, education, alcohol, smoking history, 1st degree relative with BC, age at menarche, age at first live birth, GRS, biopsy
Li, 2012	Sweden	BC risk	General	Swedish national registry	BMI, alcohol, family history of BC, age at menarche, age of menopause, age at first live birth, parity, HRT, mammographic density, age at mammogram, age at first BC diagnosis
Li, 2018 (ER+ overall)	Ten European countries	BC risk	General	European Prospective Investigation into Cancer and Nutrition cohort	Country, BMI, height, alcohol, age at menarche, age of menopause, age at first live birth, parity, breastfeeding, HRT, interaction between BMI and menopausal status
Li, 2018 (ER- overall)					
Li, 2018 (overall omnibus)					
Li, 2018 (pre-menopausal ER+)					
Li, 2018 (pre-menopausal ER-)					
Li, 2018 (pre-menopausal omnibus)					
Li, 2018 (post-menopausal ER+)					
Li, 2018 (post-menopausal ER-)					
Li, 2018 (post-menopausal omnibus)					
Li, 2018	China	Short term BC risk	General	Medical records	Mammography (global- and local-based bilateral asymmetry), mammographic density
Listgarten, 2004	Canada	BC risk	General	Interviews, medical records	SNPs
Lophatananon, 2017	UK	10-year BC risk	General	UK biobank	Alcohol, 1st degree relative with BC, age at menarche, age of menopause, parity, birth weight, HRT, OCP, benign breast disease, multivitamin supplement, obesity
Louro, 2021	Spain	BC risk	General	Medical records	Age, family history of BC, 1st degree relative with BC, mammographic density, benign breast disease
Maas, 2016 (questionnaire only)	USA	10-year BC risk	General	Breast and Prostate Cancer Cohort Consortium (BPC3) and 2010 National Health Interview Survey	BMI, height, alcohol, smoking history, family history of BC, age of menopause, menopausal status, age at first live birth, parity, HRT
Maas, 2016 (PRS-92)					PRS-92

Maas, 2016 (questionnaire + PRS-92)					BMI, height, alcohol, smoking history, family history of BC, age of menopause, menopausal status, age at first live birth, parity, HRT, PRS-92
Machiela, 2011	USA	BC risk	General	Nurses' health study	Genetic information
Marchand, 2020	USA	Postmenopausal BC risk	General	Patients in the Multiethnic Cohort (MEC)	Visceral adipose tissue biomarkers
Mavaddat, 2015	European	BC risk	General	Breast Cancer Association Consortium (BCAC)	SNPs
Mavaddat, 2019 (77 SNPs)	UK	BC risk	General	Breast Cancer Association Consortium (BCAC)	SNPs
Mavaddat, 2019 (313 SNPs)					
Mavaddat, 2019 (3820 SNPs)					
Ming, 2019 (ML random forest)	USA	BC risk	General	Medical records	Age, ethnicity, 1st degree relative with BC, age at menarche, age of first live birth, biopsy, number of biopsies, atypical hyperplasia
Ming, 2019 (ML random forest)					Age, gender, ethnicity, family history of BC, BRCA1/2, deceased status, age at first BC diagnosis, age at contralateral BC diagnosis, ER status, PR status
Ming, 2020 (ML random forest)	Switzerland	BC risk	General	Medical records	Age, ethnicity, family history of BC, BRCA1/2, vital status, history of ovarian cancer, age at ovarian cancer diagnosis, age at contralateral BC diagnosis, ER status, PR status, HER2 status
Ming, 2020 (ML adaptive boosting					
Ming, 2020 (Markov chain Monte-Carlo)					
Ming, 2020 (ML- random forest)					
Mirniaharikandehei, 2018	USA	BC risk	General	Medical records	Mammographic density
Nguyen, 2021	Australia	BC risk	General	The Melbourne Collaborative Cohort Study (MCCS), the Australian Breast Cancer Family Study, and the Australian Mammographic Density Twins and Sisters Study	Mammography (brightness and texture)

Oze, 2021 (Genetic + environmental model)	Japan	BC risk	General	Questionnaire, medical records	Age, BMI, alcohol, smoking history, family history of BC, age at menarche, age at first live birth, parity, breastfeeding, HRT, physical activity, physical activity, genetic information
Oze, 2021 (Genetic model)					Genetic information
Oze, 2021 (Environmental model)					Age, BMI, alcohol, smoking history, family history of BC, age at menarche, age at first live birth, parity, breastfeeding, HRT, physical activity, physical activity
Palmer, 2021	USA	5-year BC risk	General	Breast Cancer Surveillance Consortium (BCSC)	BMI, height, alcohol, 1st degree relative with BC, age at menarche, age at menopause, age at first live birth, HRT, benign breast disease, biopsy, 1st degree relative with ovarian cancer, diabetes mellitus type 2, bilateral oophorectomy, history of colon and prostate cancer
Pankratz, 2015	USA	5-year, 10-year, and lifetime BC risk	High risk: women with benign breast disease	Mayo benign breast disease cohort	Family history of BC, age at menarche, age at first live birth, parity, benign breast disease, biopsy histology (overall histologic impression, atypical hyperplasia if present, sclerosing adenosis/columnar alterations, extent of lobular invasion; radial scars)
Petracci, 2011	Italy	BC risk	General	Questionnaire, medical records	Age, socioeconomic status, education, BMI, alcohol, 1st degree relative with BC, age at menarche, age at first live birth, biopsy, number of biopsies, physical activity
Pfeiffer, 2013	USA	BC risk	General	Prostate, Lung, Colorectal, and Ovarian Cancer Screening Trial [PLCO], the National Institutes of Health–AARP Diet and Health Study [NIH-AARP], Nurses' Health Study cohort	BMI, alcohol, family history of BC, age of menopause, menopausal status, parity, HRT, benign breast disease, biopsy, family history of ovarian cancer
Prosperi, 2014	UK	BC risk	High risk: BRCA 1/2 carriers	Medical records	Age, BMI, age at menarche, age of menopause, age at first live birth, parity, OCP, BRCA1/2, history of oophorectomy, history of mastectomy
Qian, 2020	China	5-year and 10-year of second primary BC risk	High risk: BC survivors	Surveillance, Epidemiology and End Results (SEER) database	Age, ethnicity, tumor size, hormone receptor, histology, localized tumor, history of previous breast surgery, radiotherapy
Qiu, 2020	China	BC risk	General	Medical records	11 autoantibodies that react with tumor-associated antigens (TAAs)

Rice, 2017	USA	BC risk	General	NHS/NHSII breast cancer case-control studies	Age, BMI, height, alcohol, family history of BC, 1st degree relative with BC, age at menarche, age at first live birth, parity, HRT, mammographic density, benign breast disease, biopsy, adolescent somatotype, duration of menopause
Rosner, 2021 (age only)	USA	10-year BC risk	General	Questionnaire, medical records	Age
Rosner, 2021 (age + percentage density)					Age, mammographic density
Rosner, 2021 2021 (age + percentage density + questionnaire)					Age, BMI, height, alcohol, 1st degree relative with BC, age of first live birth, HRT, mammographic density, genetic information, benign breast disease, duration of menopause
Rosner, 2021 (age + percentage density + questionnaire + PRS)					Age, BMI, height, alcohol, 1st degree relative with BC, age of first live birth, HRT, mammographic density, genetic information, benign breast disease, duration of menopause, PRS
Sepandi, 2018	Iran	BC risk	General	Medical records	Age, family history of BC, age at menarche, parity, OCP, mammographic density, history of breast surgery, marital status, occupation
Shieh, 2020	USA (Latin American)	BC risk	General	SFBCS, NC-BCFR, RPGEH, MEC study, CAMA study, COLUMBUS-Colombia, COLUMBUS-Mexico, , Peruvian cancer center, COH/CCGCRN, and Columbus sub-studies- *see paper's data supplement for details*	SNPs
Stark, 2019	USA	5-year BC risk	General	The Prostate, Lung, Colorectal and Ovarian (PLCO) Cancer Screening Trial	Age, ethnicity, BMI, smoking history, family history of BC, 1st degree relative with BC, age at menarche, age at menopause, age at first live birth, parity, HRT, OCP, history of cancer
Stone, 2010 (mammographic density- dense + nondense area)	UK	BC risk	General	UK National Health Service Breast Cancer Screening Program in Cambridge	Mammographic density
Stone, 2010 (mammographic density- dense +					

percentage dense area)					
Stone, 2010 (mammographic density- non-dense + percentage dense area)					
Sueta, 2012 (Model with risk scores + genetic scores)	Japan	BC risk	General	Medical records	Age, BMI, family history of BC, age at menarche, age at menopause, age at first live birth, parity, physical activity, referral pattern to hospital, GRS
Sueta, 2012 (Model with risk scores only)					Age, BMI, family history of BC, age at menarche, age at menopause, age at first live birth, parity, physical activity, referral pattern to hospital
Sueta, 2012 (Model with genetic scores only)					GRS
Tan, 2013	USA	Short-term BC risk	General	Medical records	Age, family history of BC, mammographic density
Tice, 2008	USA	BC risk	General	Breast Cancer Surveillance Consortium	Age, ethnicity, family history of BC, mammographic density, biopsy
Tice, 2015 (BCSC BBD)	USA	BC risk	General	Breast Cancer Surveillance Consortium (BCSC)	Age, family history of BC, ethnicity, history of prior biopsies
Tice, 2015 (BCSC breast density model)					Age, family history of BC, ethnicity, history of prior biopsies, breast density
Tong, 2021	USA	10-year contralateral BC risk	High risk: previous BC	The Surveillance, Epidemiology, and End Results (SEER)	Age, biopsy, type of first BC, tumor size, type of previous surgery, ER/PR status, lymph nodes
Usher-Smith, 2019	UK	10-year BC risk	General	Questionnaire	BMI, alcohol, breastfeeding, HRT,
Vachon, 2015, (PRS on SERMs)	USA	BC risk	High risk: on Tamoxifen/Raloxifene	NSABP P-1 and P-2 trials	GRS
Vachon, 2015, (PRS with family history on SERMs)					Age at menarche, GRS
Van Veen, 2018 (SNPs)	UK	10-year BC risk	General	Medical records	SNPs
Van Veen, 2018 (SNPs + mammography)					SNPs, mammographic density
Wang, 2014	China	5-year BC risk	General	Medical records	Age, family history of BC, age at menarche, breastfeeding, benign breast disease, history of induced abortion

Wang, 2016 (LASSO-model for premenopausal)	Hong Kong	BC risk	General	Medical records	Age, alcohol, 1st degree relative with BC, parity, light at night, sleep quality
Wang, 2016 (LASSO-model for post-menopausal			General		Age, BMI, 1st degree relative with BC, age at menarche, age at first live birth, parity, breastfeeding, HRT, OCP, benign breast disease, light at night, sleep quality
Wang, 2016 (OPT-model for premenopausal)			General		Age, 1st degree relative with BC, age at menarche, age at first live birth, benign breast disease
Wang, 2016 (OPT-model for post-menopausal)			General		Age, 1st degree relative with BC, age at menarche, age at first live birth, benign breast disease
Wang, 2018	UK	BC risk	General	Medical records	Mammographic density
Wang, 2018	Nigeria	BC risk	General	Nigerian Breast Cancer Study	Age, BMI, height, alcohol, family history of BC, age at menarche, parity, breastfeeding, benign breast disease
Wang, 2019	China	BC risk	General	Medical records	Age, BMI, height, family history of BC, age at first live birth, benign breast disease, number of abortions, life satisfaction scores
Wacholder, 2010 (Demographic model)	USA	BC risk	General	The Nurses' Health Study, the Polish Breast Cancer Study	Age
Wacholder, 2010 (Nongenetic model)					Age, ethnicity, 1st degree relative with BC, age at menarche, age at first live birth, benign breast disease, biopsy, atypical hyperplasia
Wacholder, 2010 (Combined model)					Age, ethnicity, 1st degree relative with BC, age at menarche, age at first live birth, benign breast disease, biopsy, atypical hyperplasia, SNPs
Wu, 2014 (Bayesian Network (BN) trained SNP + mammographic model)	USA	BC risk	General	Medical records	SNPs, mammographic density
Wu, 2014 (Bayesian Network (BN) trained SNP model)					SNPs
Wu, 2014 (Bayesian Network (BN) trained mammographic model)					Mammographic density

Yala, 2021	USA, Sweden, Taiwan	1-year to 5-year BC risk	General	Medical records	Mammographic density
Yan, 2018	Unspecified	BC risk	General	Imaging	Mammographic density
Yiangou, 2021 (SNP15)	Greece	BC risk	General	MASTOS study	SNPs
Yiangou, 2021 (SNP15 + Clinical risk factor)					Age, BMI, smoking history, 1st degree relative with BC, age at menarche, age at menopause, age at first live birth, parity, breastfeeding, HRT, SNPs
Yoshimoto, 2011 (Model for ER positive premenopausal women)	Japan	BC risk	General	Medical records	Age, alcohol, parity, breastfeeding, serum levels of testosterone, serum level of prolactin, ER status
Yoshimoto, 2011 (Model for ER positive postmenopausal women)					Age, alcohol, parity, age at menopause, serum levels of testosterone, serum level of prolactin, ER status, serum level of IGF1
Zhao, 2021	China	BC risk	General	Medical records	SNPs
Zheng, 2010	China	10-year BC risk	General	Shanghai Breast Cancer Study (SBCS)	Age, WHR, family history of BC, age at menarche, age at menopause, age at first live birth, SNPs, benign breast disease
Zhu, 2021	USA	BC risk	General	Breast Cancer Surveillance Consortium (BCSC)	Age, ethnicity, BMI, family history of BC, mammographic density (Clinical BI-RADS), biopsy

Table ST2. Risk factors considered across all 106 included studies and grouped into categories. A total of 112 factors included in risk prediction models were identified across all studies. Abbreviations: BC=breast cancer.

Demographics	Reproductive history and characteristics	Social history
Sex	Age at menarche	Alcohol intake
Age	Menstrual cycle length	Smoking history
Body mass index (BMI)	Age at first live birth	Physical activity
Ethnicity	Age at subsequent births	Diet
Socioeconomic status	Pregnancy length	Multivitamin supplement
Marital status	Parity	Sleep quality
Waist-to-height ratio (WHtR)	Breastfeeding	Life satisfaction scores
Waist-to)-hip ratio (WHR)	Birth weight	Depressive symptoms
Height	History of induced abortion	Exposure to chest radiation
Residence area	Number of abortions	Past medical history
Education	Age at menopause	Referral pattern to hospital
Marital status	Menopausal status	**Genetic information**
Occupation	Duration of menopause	Single nucleotide polymorphisms (SNPs)
Family income	Duration of reproductive life span	Breast cancer gene (BRCA1 and BRCA2) status
Obesity	Serum levels of testosterone	Checkpoint kinase 2 (CHEK2) gene mutation
History of cancer	Serum of level of prolactin	Oestrogen / progesterone receptor status
History of colon and prostate cancer	Hormone replacement therapy (HRT)	Human epidermal growth factor receptor 2 (HER2) status
Type 2 diabetes mellitus	Combined oral contraceptive pill (COCP)	Immune genes
Diseased status	Oestrogen use	Zinc Finger Proteins (ZNFs)
**Family history**	Combined oestrogen and progestogen use	Adolescent somatotype
Family history of BC	History of ovarian cancer	CYP1A1, CYP1B1 and COMT genotypes
1st degree relative with BC	Age at ovarian cancer diagnosis	Transcriptional profiles of FFPE biopsy-derived RNA
Laterality of cancer in family	History of hysterectomy/oophorectomy	ATM, CHEK2, PALB2, BARD1, BRIP1, CDH1, and NF1
Family history of ovarian cancer	Bilateral oophorectomy	Genetic variants (TOX3-rs3803662, ESR1-rs2046210, 8q24-rs13281615, and SLC4A7-rs4973768)
1st degree relative with ovarian cancer	**Imaging and procedural investigations**	
Family history of male BC	Mammographic density	
**Breast cancer history**	Age at mammogram	
Benign breast disease	Ultrasound density	
History of previous BC	Thermography imaging	
Age at first BC diagnosis	Biopsy	
Type of first BC	Age at biopsy	
High risk pre-neoplasia status	Number of biopsies	
Localized tumour	Tumour size	
Chemotherapy	Histology	
Radiotherapy	Morphology	
Steroid therapy	Nuclear and histologic grade	
Adjuvant endocrine therapy	Atypical hyperplasia	
Adjuvant trastuzumab therapy	Lymphovascular invasion	

Anti-oestrogen therapy	Node-positive ratio	
History of breast surgery	Nodal status	
Type of previous surgery	Nodal size	
History of mastectomy	Serum level of insulin-like growth factor 1 (IGF-1)	
Prior breast procedure	Serum level of 25-hydroxyvitamin D	
	Hyperlipidaemia	
	% Calories from SFAs	
	% Calories from fat	
	Oncotype Dx test	
	Visceral adipose tissue biomarkers	
	Visceral adipose tissue biomarkers	

Table ST3. Summary of sample size, discriminative power, and calibration of models included across all 107 studies in this review. Discrimination is reported as the area under the receiver operating curve (AUC) and its 95% confidence interval (95% CI) unless otherwise stated. '*See paper'* indicates results were too lengthy to be reported in the table. Abbreviations: BC=breast cancer; PRS=polygenic risk scores; HRT=hormone replacement therapy; O/E=observed over expected ratio; E/O=expected over observed ratio; 95% CI=95% confidence interval; SD=standard deviation; OR=odds ratio; HR= hazard ratio; RR=relative risk; Epi=epidemiological; SNPs=single nucleotide polymorphisms; ER+=oestrogen receptor positive breast cancer; ER-=oestrogen receptor negative breast cancer; BI-RADS=breast imaging reporting and data systems; DL=deep learning.

**Development of risk score**			
**First author, year**	**Sample size**	**Measure of discrimination (95%CI)**	**Calibration**
Abdolell, 2020 (imaging only)	1,882 cases, 5,888 controls	0.597 (0.583-0.612)	No
Abdolell, 2020 (imaging + core biopsy)		0.660 (0.646-0.675)	
Abdolell, 2020 (imaging + core biopsy + family history)		0.664 (0.650-0.678)	
Abdolell, 2020 (all factors)		0.665 (0.650-0.679)	
Ahmed, 2019	404 malignant biopsies, 229 benign biopsies	0.877 (0.851-0.903)	No
Antoniou, 2002	1,484 cases, 156 controls	*Not reported*	No
Arefan, 2020	113 cases, 113 controls	0.73 (0.68-0.78)	No
Babiker, 2020	135 case, 270 controls	*See paper*	No
Banegas, 2017 (US-born)	1,086 cases, 1,411 controls	0.564 (0.485-0.644)	No
Banegas, 2017 (foreign-born)		0.625 (0.487-0.764)	
Behravan, 2020	445 cases, 250 controls	Mean average precision=77.78	No
Boggs, 2015 (<50 years old)	55,093	0.62 (0.58-0.65)	No
Boggs, 2015 (≥50 years old)		0.56 (0.53-0.59)	
Borde, 2021	760	HR=1.71 (1.36-2.15) per SD of PRS	No
Bryan, 2018 (whole genome linear mixed model)	2,109 cases, 967 controls	0.636 (0.614-0.659)	No
Bryan, 2018 (polygenic risk score)		0.601 (0.578-0.623)	
Bryan, 2018 (non-genetic epi risk factors)		0.609 (0.587–0.632	
Bryan, 2018 (non-genetic epi risk factors + LMM)		0.662 (0.640-0.684)	
Chan, 2018 (Model 1)	1595 cases, 1128 controls	0.572 (0.532-0.620)	No
Chan, 2018 (Model 2)		0.565 (0.516-0.6130)	
Chan, 2018 (Model 3)		0.557 (0.508-0.606)	
Chen, 2006 (<50 years old)	1744	0.779 (0.733-0.819)	Age-specific concordance statistics=64.3%
Chen, 2006 (≥50 years old)		0.747 (0.702-0.788)	
Chowdhury, 2017	1,921 cases, 75,825 controls	*See paper*	No

Colditz, 1996	89,132	*See paper*	No
Crooke, 2011 (4-OHE2 model)	GENICA study: 967 cases, 971 controls; Nashville Breast Cohort: 465 cases, 885 controls	0.588 (0.56-0.62)	No
Dai et al, 2012	1,792 cases, 1,867 controls	0.658 (0.640-0.676)	No
Dankova, 2019	171 cases, 146 controls	0.728	70.6% sensitivity and 65.1% specificity
Darabi, 2012	1,569 cases, 1,730 controls	0.602 (0.584-0.621)	Brier scores showed lack of fit
Dembrower, 2020 (age-adjusted DL risk score)	2,283	OR=1.56 (1.48-1.64)	No
Dembrower, 2020 (dense area and percentage density score)		OR=1.31 (1.24-1.38), AUC=0.60	
Eriksson, 2017	433 cases, 1732 controls	0.71 (0.69-0.73)	No
Evans, 2017	364 cases, 1605 controls	0.59 (0.55-0.63	*See paper*
Feld, 2018	768	0.753 (0.719-0.787)	No
Gail, 1989	2,852 cases, 3,146 controls	*See paper*	No
Gao, 2021	26,798 cases, 26,127 controls	1 SD difference in PRS associated with a 1.63-fold change (1.55-1.71) in BC risk	No
Giardiello, 2019 (PredictCBC1A)	132,756	5 years: 0.63 (0.58-0.67); 10 years: 0.63 (0.59-0.66)	Calibration-in-the-large=-0.13 (-0.66–0.40). Calibration slope=0.90 (95% CI: 0.79-1.02) in the cross-validation
Giardiello, 2019 (PredictCBC1B)		5 years: 0.59 (0.54-0.63); at 10 years: 0.59 (0.56-0.62)	Calibration-in-the-large=-0.17 (-0.72-0.38). Calibration slope=0.81 (0.63-0.99)
Gorla, 2005	1,263	*See paper*	No
Hajiloo, 2013	348 cases, 348 controls	Accuracy=59.55%	Precision=50.40%
Han, 2021 (CKB)	2,287	0.634 (0.608-0.661)	O/E=1.01 (0.94–1.09)
Han, 2021 (SWHS)		0.585 (0.564-0.605)	
He, 2012	3,683 cases, 34,174 controls	*See paper*	No
Hippisley-Cox, 2015	41,315	0.761 (0.758 to 0.765)	*See paper*
Hou, 2020 (XGBoost)	7127 cases, 7127 controls	0.742 (0.733-0.751)	No
Hou, 2020 (Deep neural network)		0.728 (0.713-0.737)	
Hou, 2020 (random forest)		0.728 (0.718-0.738)	
Hou, 2020 (logistic regression)		0.621 (0.613-0.629)	
Ho, 2021	22,013 cases, 22,114 controls	0.635 (0.622-0.649)	No
Hsieh, 2017 (PRS)	446 cases, 514 controls	0.665	*Validation executed but no results reported*
Hsieh, 2017 (PRS + clinical risk factors)		0.634	
Hughes, 2020	24,259	*See paper*	Validation 1 OR: 1.45 (1.39-1.52); validation 2 OR : 1.47 (1.45-1.49)

Hurson, 2021 (classical risk factors, <50 years old)	239,340	0.559 (0.538-0.580)	E/O=0.9 (0.7-1.0) in women <50yrs; E/O=1.0 (0.7-1.3) in women ≥50 y/o
Hurson, 2021 (integrated model, <50 years old)		0.640 (0.620-0.660)	
Hurson, 2021 (classical risk factors model, ≥50 years old)		0.574 (0.566-0.582)	
Hurson, 2021 (integrated model, ≥50 years old)		0.639 (0.632-0.647)	
Husings, 2012 (epi risk factors)	6009 cases, 7827 controls	0.564 (0.547-0.581)	No
Husings, 2012 (18 SNPs)		0.584 (0.567-0.600)	
Husings, 2012 (epi risk factors + GWAS)		0.605 (0.589-0.622)	
Jiang, 2019 (model 0)	37,272	0.593	GOF = 3.16×10^{-14}
Jiang, 2019 (model 1a)		0.578	GOF = 1.22×10^{-9}
Jiang, 2019 (model 1b)		0.580	GOF = 1.14×10^{-7}
Jiang, 2019 (model 2a)		0.594	GOF = 1.16×10^{-11}
Jiang, 2019 (model 2b)		0.596	GOF = 4.47×10^{-9}
Jiang, 2019 (model 3a)		0.596	GOF = 0.0213
Jiang, 2019 (model 3b)		0.697	GOF = 0.1108
Jung, 2018	352 cases, 6215 controls	*See paper*	No
Kakileti, 2020 (77SNPs)	769	0.895	No
Kerlikowske, 2015 (Two BIRADs 5-year risk)	722,654	0.64	E/O=0.98 (0.96-1.00)
Kerlikowske, 2015 (One BIRADs 5-year risk)		0.635	E/O=0.98 (0.96-1.00)
Kerlikowske, 2015 (Two BIRADs 10-year risk)		0.628	E/O=0.95 (0.94-0.96
Kerlikowske, 2015 (One BIRADs 10-year risk)		0.622	E/O=0.95 (0.94-0.96)
Kerlikowske, 2018 (interval BC)	1609 with screen-detected cancer, 351 with interval invasive cancer, 4409 matched controls	C-statistic=0.70 vs. 0.62 (P< 0.001) for automated and clinical BI-RADS density	No
Kerlikowske, 2018 (screen-detected BC)		C-statistic=0.72 vs. 0.62 (P< 0.001) for automated and clinical BI-RADS density	
Kerlikowske, 2022	931,186	0.682 (0.670-0.694)	Annual screening=E/O 1.00 (0.94-1.06); for biennial screening=E/O 1.00 (0.93-1.08)
Kuchenbaecker, 2017 (BRCA1)	23,463	C-statistic=0.541 (0.530-0.551)	No
Kuchenbaecker, 2017 (BRCA2)		C-statistic=0.566 (0.551-0.581)	
Kuchenbaecker, 2017 (BRCA1, ER+ BC)		C-statistic=0.532 (0.522-0.543)	
Kuchenbaecker, 2017 (BRCA2, ER+ BC)		C-statistic=0.566 (0.551-0.581)	
Kuchenbaecker, 2017 (BRCA1, ER- BC)		C-statistic=0.581 (0.571-0.592)	
Kuchenbaecker, 2017 (BRCA2, ER- BC)		C-statistic=0.538 (0.523-0.553)	
Lacaze, 2021 (PRS)	6,339	0.62 (0.59-0.65)	No
Lacaze, 2021 (BC only)		0.53 (0.52-0.55)	
Läll, 2019	32,557	*See paper*	No

Lecarpentier, 2017 (ER+ PRS)	277 cases, 1,313 controls	0.59 (0.55-0.63)	No
Lecarpentier, 2017 (ER- PRS)		0.55 (0.51-0.59)	
Lee, 2004	384 cases; 166 controls	0.695	0.714 (no 95%CI)
Lee, 2014	411 cases, 1212 controls	NRI=3.4% (p=0.006)	NRI decreased to 6.2% after correcting for optimism using a bootstrap method
Li, 2012	733 cases, 748 controls	0.589 (0.561-0.618)	No
Li, 2018 (ER+ overall)	281,330	0.68 (0.65-0.70)	E/O=1.10 (1.05-1.14)
Li, 2018 (ER- overall)		0.68 (0.64-0.72)	
Li, 2018 (overall omnibus)		0.68 (0.66-0.70)	
Li, 2018 (pre-menopausal ER+)		0.64 (0.59-0.68)	
Li, 2018 (pre-menopausal ER-)		0.58 (0.51-0.66	
Li, 2018 (pre-menopausal omnibus)		0.62 (0.59-0.66	
Li, 2018 (post-menopausal ER+)		0.62 (0.59-0.66	
Li, 2018 (post-menopausal ER-)		0.60 (0.52-0.67)	
Li, 2018 (post-menopausal omnibus)		0.62 (0.59-0.65)	
Li, 2018	566 cases, 283 controls	0.687 (0.643-0.729)	No
Listgarten, 2004	174 cases, 158 controls	*See paper*	*See paper*
Lophatananon, 2017 (controls with no cancer)	3,378 cases; 235,603 non-cancer controls; 59,731 healthy controls	0.58 (0.57-0.60)	Calibration curves for both comparisons also suggested both models calibrated well
Lophatananon, 2017 (healthy controls)		0.64 (0.63-0.66)	
Maas, 2016 (questionnaire-based risk factors only)	17,171 cases, 19,862 controls	0.588	No
Maas, 2016 (PRS-92)		0.623	
Maas, 2016 (questionnaire based risk factors + PRS-92)		0.648	
Machiela, 2021	1145 cases, 1142 controls	0.59 for entire dataset; 0.50-0.53 using cross-validation subsets	No
Marchand, 2020	950 cases, 950 controls	*See paper*	No
Mavaddat, 2015	67,054	0.615 (0.608-0.616)	No
Ming, 2019 (ML adaptive boosting, US)	3,624	0.883 (0.654-1.112)	No
Ming, 2019 (ML random forest, US)		0.889 (0.869-0.909)	
Ming, 2019 (ML adaptive boosting, Swiss)		0.902 (0.886-0.918)	
Ming, 2019 (ML random forest, Swiss)		0.893 (0.239-1.547)	
Ming, 2020 (ML adaptive boosting)	45,110	0.889 (0.885-0.903)	No
Ming, 2020 (ML Markov Chain Monte-Carlo)		0.851 (0.847-0.856)	
Ming, 2020 (ML random forest)		0.843 (0.838-0.849)	
Mirniaharikandehei, 2018 (craniocaudal images)	1,044	0.586 (0.550-0.621)	No
Mirniaharikandehei, 2018 (mediolateral oblique images)		0.652 (0.617-0.686)	
Nguyen, 2021 (interval BC)	944 cases, 2,639 controls	0.73	No
Nguyen, 2021 (screen-detected BC)		0.63	
Nguyen, 2021 (younger BC diagnosis)		0.72	

Oze, 2021 (genetic + environmental factors)	1,319 cases, 2,094 controls	C-statistic=0.659 (0.640–0.678)	Calibration slope of 1.02 and p-value for Hosmer–Lemeshow test=0.506
Oze, 2021 (genetic factors only)		C-statistic=0.633 (0.614- 0.652)	No
Oze, 2021 (environmental factors only)		C-statistic=0.616 (0.596–0.636)	No
Prosperi, 2014 (BRCA1)	1,071	C-statistic (SD)=0.779	No
Prosperi, 2014 (BRCA2)		C-statistic (SD)= 0.785	
Qian, 2020	208,474	C-index=0.61	No
Rice, 2017	2,955	OR=1.09 (1.07-1.11)	No
Sepandi, 2018	655	0.955	Sensitivity=0.82; specificity=0.90
Shieh, 2020	13,624	0.63 (0.62-0.64)	Two-sided Hosmer-Lemeshow: X^2=10.45, p=0.32 for 180-SNP PRS
Stark, 2019	64,739	0.613 (0.579-0.647)	No
Sueta, 2012 (risk score + genetic risk score)	697 cases, 1394 controls	0.693	No
Sueta, 2012 (risk score only)		0.665	
Sueta, 2012 (genetic score only)		0.597	
Tan, 2013	994	0.725 (0689-0.759)	PPV=71.3%
Tice, 2008	1,095,484	0.66 (0.65-0.67)	E/O=1.03 (0.99 to 1.06)
Tice, 2015 (BCSC BBD model)	1,135,977	0.665	E/O=1.04 (1.03-1.06)
Tice, 2015 (BCSC breast density model)		0.664	E/O=0.86
Tong, 2021	434,065	0.65	*Calibration as per figure in paper*
Vachon, 2015 (BI-RADS only)	1,763	0.66 (0.64-0.68)	No
Vachon, 2015 (quartile PRS only)		0.68 (0.66-0.69)	
Vachon, 2015 (BI-RADS + quartile PRS)		0.69 (0.67-0.70)	
Vachon, 2015 (BI-RADS + continuous PRS)		0.69 (0.67-0.71)	
Van Vee, 2018 (SNPs)	8,897 cases, 466 controls	OR=1.56 (1.38-1.77)	O/E OR=1.03 (0.74-1.32)
Van Vee, 2018 (SNPs + mammography)		OR=1.53 (1.35-1.74)	O/E OR=0.98 (0.69-1.28)
Wang, 2014	918 cases, 923 controls	0.64 (0.50-0.78)	No
Wang, 2016 (Model 1)	1,841	0.640 (0.598-0.681)	No
Wang, 2016 (Model 2)		0.655 (0.621-0.653)	
Wang, 2018	First group=1264 (317 cases, 947 controls) second group=1253 (318 cases, 935 controls)	Akaike information criterion (AIC)=5.90	No
Wacholder, 2010 (demographics)	5,590 cases, 5,998 controls	0.534	No
Wacholder, 2010 (non-genetic)		0.580	
Wacholder, 2010 (combined)		0.618	
Wu, 2014 (SNPs + mammographic density)	373 cases, 395 controls	0.736	No
Wu, 2014 (trained SNPs model)		0.581	
Wu, 2014 (trained mammographic model)		0.704	
Yala, 2021 (MCH)	25,855	0.76 (0.74-0.80)	No
Yala, 2021 (Karolinska University model)	19,328	0.81 (0.79-80.2)	
Yala, 2021 (CGMH model)	13,356	0.79 (0.79-0.83)	
Yan, 2018	280 cases, 280 controls	0.898 (0.885-0.911)	No

Yiangou, 2021 (SNP15)	1,109 cases, 1,177 controls	0.55 (0.52-0.57)	Hosmer–Lemeshow test x^2= 11.77; p-value= 0.162
Yiangou, 2021 (SNP15 + clinical risk factors)		0.70 (0.67-0.72)	Hosmer–Lemeshow test x^2=8.73; p-value=0.37
Yoshimoto, 2011 (ER+ premenopausal women)	535	0.848	No
Yoshimoto, 2011 (ER+ postmenopausal women)		0.772	
Zhao, 2021 (Model 1)	Estrogen levels: 64 cases, 49 controls. Genotype: 140 cases, 140 controls	0.546	No
Zhao, 2021 (Model 2)		0.622	
Zheng, 2010	3,039 cases, 3,082 controls	0.623	No
Zhu, 2021 (DL model)	6,369	0.66 (0.63, 0.69)	No
Zhu, 2021 (clinical risk factors + BI-RADS)		0.62 (0.59-0.65)	
Zhu, 2021 (DL + clinical risk factors		0.66 (0.63-0.69)	

Table ST4. Summary of sample size, discriminative power, calibration, and external validation of models performing an external validation and included in this review. Discrimination is reported as the area under the receiver operating curve (AUC ROC) and associated 95% confidence intervals (95%CI) unless otherwise stated. External validation is also reported in the same manner. Abbreviations: BC=breast cancer; SNPs=single nucleotide polymorphisms; PRS=polygenic risks cores; O/E=observed over expected ratio; E/O=expected over observed ratio; 95%CI=95% confidence interval; SD=standard deviation; OR=odds ratio; HR=hazard ratio; RR=relative risk; ZNF=zinc finger-like proteins.

	Development of risk score			External validation of risk score		
**First author, year**	**Sample size**	**Discrimination, AUC (95%CI) unless otherwise stated**	**Calibration**	**Sample size**	**Discrimination (AUC and 95%CI unless stated otherwise)**	**Calibration**
Anothaisintawee, 2014	15,718	C-statistic=0.651 (0.595-0.707)	O/E ratio=1.00 (95% CI:0.82-1.21)	4,978	C statistic=0.97 (95%CI: 0.68-1.35)	O/E ratio=0.609 (95% CI: 0.511-0.706)
Barlow, 2006	426,224	0.624 (0.619-0.630)	C-statistic=0.631 (0.618-0.644) for premenopausal women and 0.624 (0.619-0.630) for postmenopausal women	141,991	0.629 (0.603-0.656)	Lack of fit
Barnes, 2020 (BRCA1)	23,463	HR=1.32 (1.25-1.40)	No	3,845	HR=1.36 (0.17-1.57)	No
Barnes, 2020 (BRCA2)		HR=1.44 (1.30–1.60)				
Chen, 2021	577	0.752	0.704	280	3- and 5-year overall survival=0.685 and 0.717, respectively (p=0.00048)	No
Danladi, 2020	318 cases, 337 controls	0.66 (0.62-0.70)	No	126 cases, 527 controls	0.69 (0.63-0.74)	No
Degnim, 2015	Twin set=86, validation=65	0.91 (0.87-0.95)	Prediction accuracy=80% (70-88)	65	0.836 (95%CI: 0.75-0.92)	Prediction accuracy=58% (46-71)
Hughes, 2021	28,928 cases, 112,232 controls	*See paper*		1615	CRS RLR OR=2.08 (1.83-2.37) in validation 1, CRS RLR OR=2.44 (1.89-3.19) in validation 2. CRS 5-year risk OR=4.58 (3.57-5.90) in validation 1, CRS 5-year risk OR= 2.46 (1.90-3.21) in validation 2	Average RLR and 5-year risk estimates matched exactly in both validations (RLR 12.6%; 5-year 0.96%)
Louro, 2021	73,181	0.647 (0.625-0.669)	E/O ratio=0.99 at 2 years; 1.02 at 20 years	48,787	*External validation performed but results not reported*	*External validation performed but results not reported*

Mavaddat, 2019 (77 SNPs)	94,075 cases, 75,017 controls	Overall BC=0.612; ER+ =0.623; ER- =0.596	No	11,428 cases, 18,323 controls	Overall BC=0.603; ER+ =0.615; ER-=0.584	No
Mavaddat, 2019 (313 SNPs)		Overall BC=0.639; ER+=0.510; ER-=0.611			Overall BC=0.639; ER+=0.651; ER-=0.611	
Mavaddat, 2019 (3,830 SNPs)		Overall BC=0.646; ER+ =0.659; ER-=0.611			Overall BC=0.636; ER+ =0.647; ER-=0.600	
Pankratz, 2015	377 cases, 734 controls	5-year risk: C-statistic=0.692 (0.62-0.77); 10-year risk: C-statistic=0.665 (0.61-0.72); lifetime risk: C-statistic=0.636 (0.60-0.67)	Hosmer-Lemeshow test: P=0.247	378 cases, 728 controls	5-year risk: C-statistic=0.644 (0.57-0.72); 10-year risk: C-statistic=0.629 (0.58-0.68); lifetime risk: C-statistic=0.650 (0.62-0.68)	No
Petracci, 2011	2,569 cases, 2,588 controls	ARR=1.6% (0.9-2.3)	E/O=1.10 (0.96-1.26)	10,083	0.62 (0.56-0.69) for <50 y/o and 0.57 (0.52-0.61) for > 50 y/o	0.160 (0.099-0.230)
Pfeiffer, 2013	240,712	*See paper*	O/E ratio=1.00 (95%CI: 0.96-1.04)	57,906	0.58 (95% CI: 0.57–0.59)	No
Qiu, 2020	184 cases, 184 healthy controls, 200 BC controls	BC vs healthy=0.943; BC vs benign breast disease=0.881	No	415	BC vs healthy=0.916; BC vs benign breast disease= 0.849	No
Rosner, 2021 (age only)	2,799 cases, 75,557 controls	0.523	No	434 cases, 898 controls	0.595	No
Rosner, 2021 (age + percentage density		0.629			0.635	
Rosner, 2021 (age + percentage density + questionnaire)		0.637			0.650	
Rosner, 2021 (age + percentage density + questionnaire + PRS)		0.658			0.687	
Palmer, 2021	6,668	0.58 (0.56 to 0.59)	O/E=1.01 (0.95-1.07)	51,798	0.56 (0.55-0.59)	0.97 (0.92-1.03)
Stone, 2010	2,514	Risk increased by 42% (29-56) per SD of square root dense area; by 37% (25-50) per SD and decreased by 11% (2-19) per SD of square root non-dense area	Two-sided Hosmer-Lemeshow χ^2=11.6, p<0.001	1,880	OR=1.00 (0.90-1.10) for sense area; OR=1.10 (0.93-1.29) for PDA	No
Usher-Smith, 2009	1,000	0.56 (0.52-0.58)	O/E=1.08 50.90-1.30), at higher risks, the models overestimated risk	25,639	*External validation performed but not reported for BC*	*External validation performed but not reported for BC*
Wang, 2018	4,036	0.703 (0.687-0.719)	O/E=1.01 (0.93-1.09)	1,344	0.694 (0.666–0.721)	No

Wang, 2019	328 cases, 656 controls	*See paper*	E/O ratio=1.03; C-statistic=0.64	13,176	0.64 (0.55-0.72)	E/O=1.03

Table ST5. Prediction Model Risk of Bias Assessment T (PROBAST) quality assesment of included studies. The positive symbol "+" indicates low risk of bias or concern regarding applicability; the negative symbol "−" indicates high risk of bias or concern regarding applicability; the question mark "?" indicates unclear risk of bias or concern regarding applicability.

Study	ROB				Applicability			Overall	
	Participants	Predictors	Outcome	Analysis	Participants	Predictors	Outcome	ROB	Applicability
Abdolell, 2020	+	+	+	+	+	+	+	+	+
Ahmed, 2019	-	-	?	-	+	+	+	-	+
Anothaisintawee, 2014	+	+	?	-	+	+	+	-	+
Antoniou, 2002	-	+	+	+	+	+	+	-	+
Arefan, 2020	-	-	+	-	-	-	+	-	-
Arthur, 2020	+	+	+	+	+	+	+	+	+
Babiker, 2020	+	-	+	-	+	+	+	-	+
Banegas, 2017	+	+	+	+	+	+	+	+	+
Barlow, 2006	+	+	+	+	+	+	+	+	+
Barnes, 2020	+	+	+	+	+	+	+	+	+
Behravan, 2020	+	+	+	+	+	+	+	+	+
Boggs, 2015	+	+	+	+	+	+	+	+	+
Borde, 2021	-	+	+	-	-	+	+	-	-
Bryan, 2018	-	+	+	-	-	+	+	-	-
Chan, 2018	-	+	+	-	-	+	+	-	-
Chen, 2006	+	+	+	+	+	+	+	+	+
Chen, 2021	+	+	+	-	+	-	+	-	+
Chowdhury, 2017	-	+	+	-	-	+	+	-	-
Colditz & Rosner, 1996	+	+	+	+	+	+	+	+	+
Crooke, 2011	+	+	+	+	+	+	+	+	+
Dai, 2012	-	+	+	-	+	+	+	-	+
Danková, 2019	+	+	+	+	+	+	+	+	+
Danladi, 2020	-	+	+	-	+	+	+	-	+
Darabi, 2012	-	+	+	+	+	+	+	+	+

Degnim, 2015	-	+	+	-	-	-	+	-	-
Dembrower, 2020	+	+	+	+	+	+	+	+	+
Eriksson, 2017	+	+	+	+	+	+	+	+	+
Evans, 2017	+	+	+	+	-	+	+	+	+
Feld, 2018	-	+	+	-	-	+	+	-	-
Gail, 1989	+	+	+	+	+	+	+	+	+
Gao, 2021	+	+	+	+	+	+	+	+	+
Giardiello, 2019	+	+	+	+	+	+	+	+	+
Gorla, 2005	+	+	+	+	+	+	+	+	+
Hajiloo, 2013	-	?	+	-	-	?	+	-	-
Han, 2021	+	+	+	+	+	+	+	+	+
He, 2012	-	+	+	-	-	+	+	-	+
Hippisley-Cox, 2015	+	+	+	+	+	+	+	+	+
Hou, 2020	+	+	+	+	+	+	+	+	+
Ho, 2022	+	+	+	+	+	+	+	+	+
Hsieh, 2017	+	+	+	+	+	+	+	+	+
Hughes, 2020	+	+	+	+	+	+	+	+	+
Hughes, 2021	-	+	+	+	-	+	+	-	-
Hurson, 2022	+	+	+	+	+	+	+	+	+
Hüsing, 2012	+	+	+	+	+	+	+	+	+
Jiang, 2019	+	+	+	+	+	+	+	+	+
Jung, 2018	+	+	+	+	+	+	+	+	+
Kakileti, 2020	-	+	+	-	-	+	+	-	+
Kerlikowske, 2015	+	+	+	+	+	+	+	+	+
Kerlikowske, 2018	+	+	+	+	+	+	+	+	+
Kerlikowske, 2022	+	+	+	+	+	+	+	+	+
Kuchenbaecker, 2017	+	+	+	+	+	+	+	+	+
Lacaze, 2021	+	+	+	+	+	+	+	+	+
Läll, 2019	+	+	+	+	+	+	+	+	+

Lecarpentier, 2017	+	+	+	+	+	+	+	+	+
Lee, 2004	+	?	-	-	+	?	-	-	-
Lee, 2014	+	+	+	+	+	+	+	+	+
Li, 2012	+	+	+	+	+	+	+	+	+
Li, 2018	+	+	+	+	+	+	+	+	+
Li, 2018	+	+	+	+	+	+	+	+	+
Listgarten, 2004	-	+	-	?	-	+	+	-	?
Lophatananon, 2017	+	+	+	+	+	+	+	+	+
Louro, 2021	+	+	+	+	+	+	+	+	+
Maas, 2016	+	+	+	+	+	+	+	+	+
Machiela, 2011	+	+	+	+	+	+	+	+	+
Marchand, 2020	+	+	+	+	+	+	+	+	+
Mavaddat, 2015	+	+	+	+	+	+	+	+	+
Mavaddat, 2019	+	+	+	+	+	+	+	+	+
Ming, 2019	+	+	+	+	+	+	+	+	+
Ming, 2020	+	+	+	+	+	+	+	+	+
Mirniaharikandehei, 2018	?	+	+	?	?	+	+	?	?
Nguyen, 2021	+	+	+	+	+	+	+	+	+
Oze, 2021	+	+	+	+	+	+	+	+	+
Palmer, 2021	+	+	+	+	+	+	+	+	+
Pankratz, 2015	+	+	+	+	+	+	+	+	+
Petracci, 2011	+	+	+	+	+	+	+	+	+
Pfeiffer, 2013	+	+	+	+	+	+	+	+	+
Prosperi, 2014	+	+	+	?	+	+	+	?	+
Qian, 2020	+	+	+	+	+	+	+	+	+
Qiu, 2020	+	+	+	+	+	+	+	+	+
Rice, 2017	+	+	+	+	+	+	+	+	+
Rosner, 2021	+	+	+	+	+	+	+	+	+
Sepandi, 2018	+	?	+	?	+	?	+	?	?

Shieh, 2020	+	+	+	+	+	+	+	+	+
Stark, 2019	-	-	-	?	-	-	-	-	-
Stone, 2010	+	+	+	+	+	+	+	+	+
Sueta, 2012	-	+	+	-	-	+	+	-	-
Tan, 2013	?	?	+	?	?	-	+	?	?
Tice, 2008	+	+	+	+	+	+	+	+	+
Tice, 2015	+	+	+	+	+	+	+	+	+
Tong, 2021	+	+	+	+	+	+	+	+	+
Usher-Smith, 2019	+	+	+	+	+	+	+	+	+
Vachon, 2015	+	+	+	?	+	+	+	?	+
Van Veen, 2018	+	+	+	+	+	+	+	+	+
Wang, 2014	+	+	+	-	+	+	+	-	+
Wang, 2016	+	+	+	?	+	+	+	?	+
Wang, 2018	+	+	+	?	+	+	+	?	+
Wang, 2018	+	+	+	+	+	+	+	+	+
Wang, 2019	+	+	+	+	+	+	+	+	+
Wacholder, 2010	+	+	+	+	+	+	+	+	+
Wu, 2014	+	+	+	+	+	+	+	+	+
Yala, 2021	+	+	+	+	+	+	+	+	+
Yan, 2018	+	+	+	?	+	+	+	?	+
Yiangou, 2021	+	+	+	+	+	+	+	+	+
Yoshimoto, 2011	+	+	+	?	+	+	+	?	+
Zhao, 2021	+	?	+	?	+	+	+	?	+
Zheng, 2010	+	+	+	?	+	+	+	+	+
Zhu, 2021	+	?	+	?	+	?	+	?	+

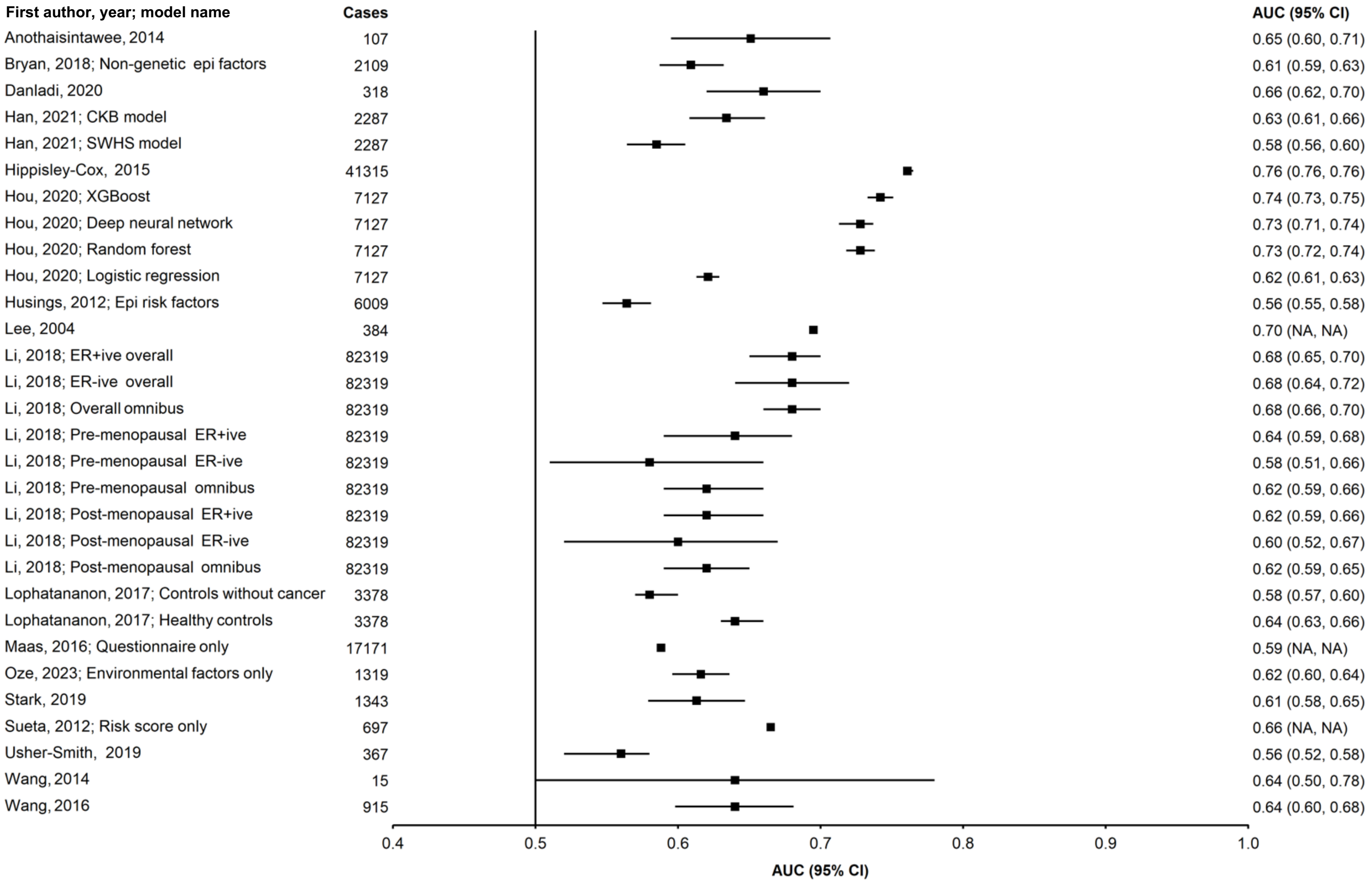

Figure SF1. Area under the receiver operating curve (AUC) and associated 95% confidence intervals (95% CIs) for all models containing demographic variables only and developed in the general population. For each study, all reported models were included in the plot. NA indicates that the data was not reported. Squares represent the AUC and line segments the 95% CIs.

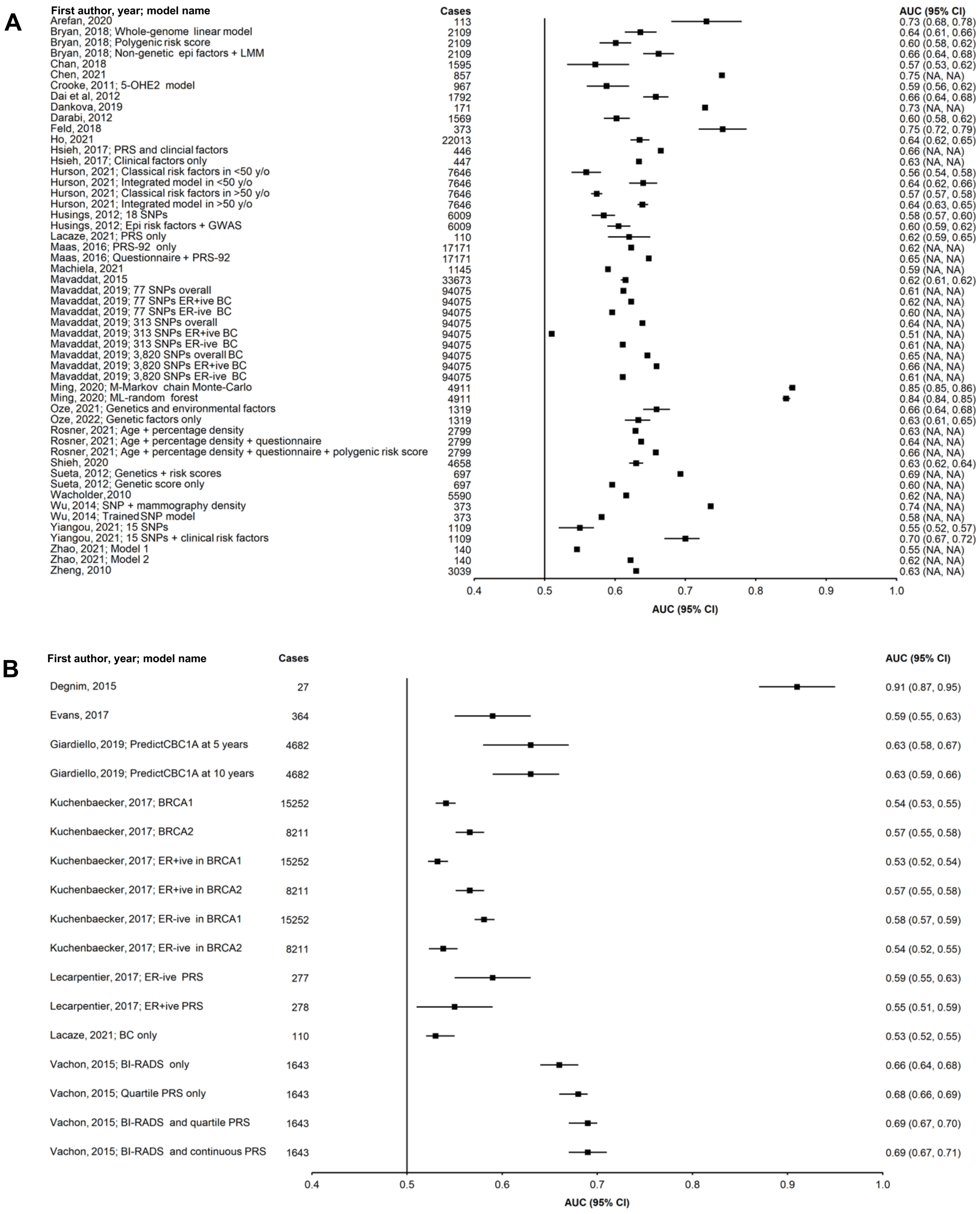

Figure SF2. Area under the receiver operating curve (AUC) and associated 95% confidence intervals (95%CIs) for models containing genetic variables and developed in general (Panel A) and high-risk (Panel B) populations. For each study, all reported models were included in the plot. NA indicates that the data was not reported. Squares represent the AUC, and the line segments represent the 95% CIs.

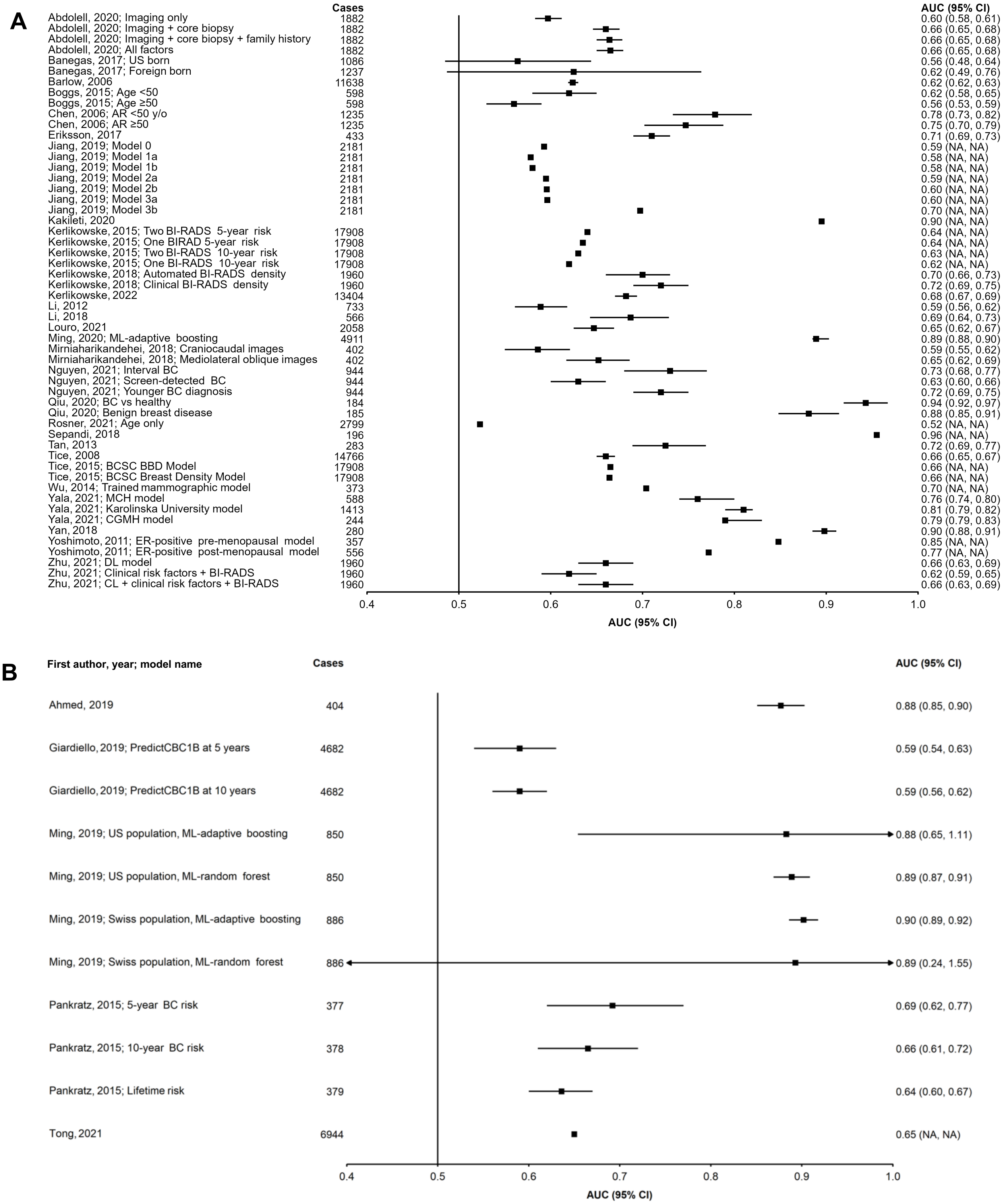

Figure SF3. Area under the receiver operating curve (AUC) and associated 95% confidence intervals (95%CIs) for models containing imaging and biopsy variables and developed in general (Panel A) and high-risk (Panel B) populations. For each study, all reported models were included in the plot. NA indicates that the data was not reported. Squares represent the AUC, and line segments represent the 95% CIs.

Supplemental Methods. Search strategy for MEDLINE and Embase.

MEDLINE search strategy:

1 exp Breast Neoplasms/
2 (breast adj2 (cancer? or carcinoma? or tumour? or tumor? or neoplas*)).
3 1 or 2
4 (risk* adj2 (predict* or estimat*)).ti,ab,kw.
5 ((risk? or prediction) adj3 (model* or score? or tool?)).
6 Risk Assessment/mt [Methods]
7 risk/ or risk assessment/ or risk factors/ or (risk* or predict*).ti.
8 models, theoretical/ or models, biological/ or models, statistical/ or (model* or scor* or tool*).
9 7 and 8
10 4 or 5 or 6 or 9
11 3 and 10
12 exp Cohort Studies/
13 exp Case Control Studies/
14 SEER Program/
15 cohort.ti,ab,kw.
16 ((case adj2 control) or ((case or control) adj2 (group? or data or subject?))).
17 nurse* health study.
18 women* health initiative.
19 (bcddp or Breast Cancer Detection Demonstration Project*).
20 (seer or "surveillance epidemiology and end results").
21 12 or 13 or 14 or 15 or 16 or 17 or 18 or 19 or 20
22 11 and 21
23 ((breast adj2 (cancer? or carcinoma? or tumour? or tumor? or neoplas*)) and (risk* or predict* or estimat*) and (model* or tool* or score*)).
24 22 or 23
25 limit 24 to english language

Embase search strategy:

1 exp *breast cancer/
2 (breast adj2 (cancer? or carcinoma? or tumour? or tumor? or neoplas*)).
3 1 or 2
4 (risk* adj2 (predict* or estimat*)).
5 ((risk? or prediction) adj3 (model* or score? or tool?)).
6 cancer risk/ or *risk/ or *risk assessment/ or *risk factor/ or (risk* or predict*).
7 *model/ or cancer model/ or (model* or scor* or tool*).
8 6 and 7
9 4 or 5 or 8
10 3 and 9

11 Cohort Analysis/
12 case control study/ or population based case control study/ or family study/ or longitudinal study/ or prospective study/ or retrospective study/
13 major clinical study/
14 cohort.
15 ((case adj2 control) or ((case or control) adj2 (group? or data or subject?))).
16 nurse* health study.
17 women* health initiative.
18 (bcddp or Breast Cancer Detection Demonstration Project*).
19 (seer or "surveillance epidemiology and end results").
20 11 or 12 or 13 or 14 or 15 or 16 or 17 or 18 or 19
21 10 and 20
23 21 not 22
24 limit 23 to english language
